# Longitudinal Functional Assessment of Brain Injury Induced by High-Intensity Ultrasound Pulse Sequences

**DOI:** 10.1038/s41598-019-51876-5

**Published:** 2019-10-29

**Authors:** Meijun Ye, Krystyna Solarana, Harmain Rafi, Shyama Patel, Marjan Nabili, Yunbo Liu, Stanley Huang, Jonathan A. N. Fisher, Victor Krauthamer, Matthew Myers, Cristin Welle

**Affiliations:** 1 Division of Biomedical Physics, Office of Science and Engineering Laboratories, Center for Devices and Radiological Health, Food and Drug Administration, Silver Spring, MD USA; 2 Division of Neurological and Physical Medicine Devices, Office of Device Evaluation, Center for Devices and Radiological Health, Food and Drug Administration, Silver Spring, MD USA; 3 Division of Applied Mechanics, Office of Science and Engineering Laboratories, Center for Devices and Radiological Health, Food and Drug Administration, Silver Spring, MD USA; 4 Division of Radiological Health, Office of In Vitro Diagnostics and Radiological Health, Center for Devices and Radiological Health, Food and Drug Administration, Silver Spring, MD USA; 5 Department of Physiology, New York Medical College, Valhalla, NY USA; 6 Departments of Neurosurgery and Physiology & Biophysics, University of Colorado Anschutz Medical Campus, Aurora, CO USA

**Keywords:** Neurophysiology, Diseases of the nervous system

## Abstract

Exposure of the brain to high-intensity stress waves creates the potential for long-term functional deficits not related to thermal or cavitational damage. Possible sources of such exposure include overpressure from blast explosions or high-intensity focused ultrasound (HIFU). While current ultrasound clinical protocols do not normally produce long-term neurological deficits, the rapid expansion of potential therapeutic applications and ultrasound pulse-train protocols highlights the importance of establishing a safety envelope beyond which therapeutic ultrasound can cause neurological deficits not detectable by standard histological assessment for thermal and cavitational damage. In this study, we assessed the neuroinflammatory response, behavioral effects, and brain micro-electrocorticographic (µECoG) signals in mice following exposure to a train of transcranial pulses above normal clinical parameters. We found that the HIFU exposure induced a mild regional neuroinflammation not localized to the primary focal site, and impaired locomotor and exploratory behavior for up to 1 month post-exposure. In addition, low frequency (δ) and high frequency (β, γ) oscillations recorded by ECoG were altered at acute and chronic time points following HIFU application. ECoG signal changes on the hemisphere ipsilateral to HIFU exposure are of greater magnitude than the contralateral hemisphere, and persist for up to three months. These results are useful for describing the upper limit of transcranial ultrasound protocols, and the neurological sequelae of injury induced by high-intensity stress waves.

## Introduction

The interaction of stress waves with neural tissue can cause changes in neural functionality that are not accompanied by morphological changes observable with post-mortem histology or current imaging modalities^1–3^. A signature feature of mild traumatic brain injury (mTBI), for example, is the presence of behavioral changes even though MRI or CT scans show no evidence of damage^4–6^. In acoustic neuromodulation, transient functional changes in the nervous system occur, enabling the transient inhibition or excitation of electrical signals (reviewed in^7^). These functional changes usually occur during the sonication, but can outlast the sonication by several minutes to 2 hours depending on the intensity and duty cycle^1–3,8–10^. While no evidence of long-term neural changes has been reported for current clinical neuromodulation procedures, the envelope outside of which long-term deficits occur has not been established. Similarly, while reports of enduring neurological deficits have been reported only for higher-intensity therapeutic ultrasound procedures resulting in lesions of specific locations and sizes^11^, general safety limits addressing the exposure of nervous tissue to intense stress waves - analogous to thermal and cavitational safety limits for sensitive tissues - have not been established.

The purpose of this study is to present evidence of long-term neurological deficits occurring during exposure of mice brains to an ultrasound pulse train, and to provide methods for quantifying and tracking the deficits. The characteristics of the ultrasound pulse train do not reflect any current clinical ultrasound procedure. Rather, the ultrasound protocol was chosen to have a high likelihood of generating neurological deficits, based upon previous reports^8,10,12^, not be accompanied by gross thermal damage or destruction due to cavitation. Identifying this critical threshold, where conventional gross histology methods may fail to detect damage, but neurological deficits persist, is critical given the growing exploration of novel therapeutic modalities and protocols for ultrasound therapies^13–19^. This study will contribute to the understanding of possible damage mediated by high-intensity pressure waves. Subsequent studies, employing the tools introduced here, could more thoroughly define the safety envelope in ultrasound parameter space. The relevant tools include a technique for measuring surface micro-electrocorticographic (µECoG) signal changes, and a method quantifying deficits in behavior involving locomotion and spontaneous exploration, following exposure to stress waves caused by ultrasound sonication in a highly controlled manner. To complement these electrophysiological and behavioral approaches, immunohistochemical measures of neuroinflammatory response, including microglial and astrocyte reactivity, are obtained in regions throughout the brain. Understanding the correlation between functional and structural responses will provide a guide in the selection of appropriate methods for safety evaluation of ultrasound while novel paradigms are been developed. Additionally, an understanding of the connection between ultrasound exposure parameters and behavioral and electrophysiological responses will improve our ability to produce brain injury for the purpose of diagnosing and treating mTBI.

## Results

### Histological assessment of neuroinflammatory responses after HIFU pulse-train exposure

**Figure 1:**
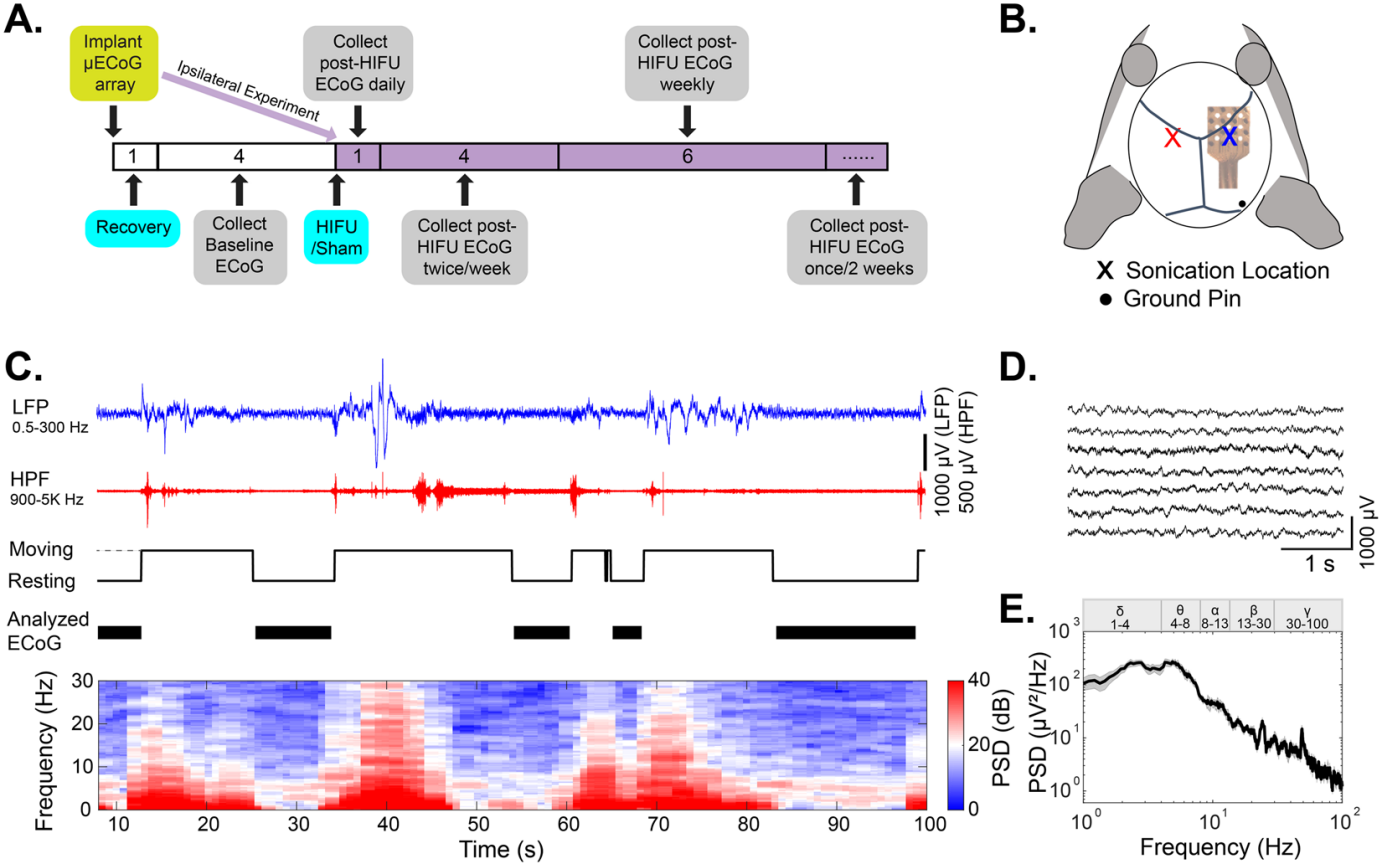
Electrophysiology experimental paradigm. (**A**) Experimental timeline. Numbers are in weeks. Ipsilateral and contralateral experiments were performed on different animal cohorts. Contralateral animals had a recovery and baseline data collection period before HIFU or sham treatment, while in ipsilateral animals, no baseline data were collected (in purple). (**B**) Illustration of µECoG array placement and sonication location. (**C**) Example raw EEG data and movement activities acquired from freely moving mice in their home cages. The presence of myoelectric and movement artifacts correlated with animal moving. Analyzed EEG signals were representative of resting state EEG, as indicated by black bars on the fourth row of the figure. The bottom heatmap shows the time-frequency analysis of raw EEG signals. Myoelectric and movement artifacts produced contamination in power spectral density (PSD) across the full frequency range, with 0–30 Hz shown in the figure. (**D**) Extracted 4 s clean EEG snippets from data shown in (**C**). (**E**) Averaged PSD of EEG signals in (D). Gray area indicates the standard error.

**Figure 2:**
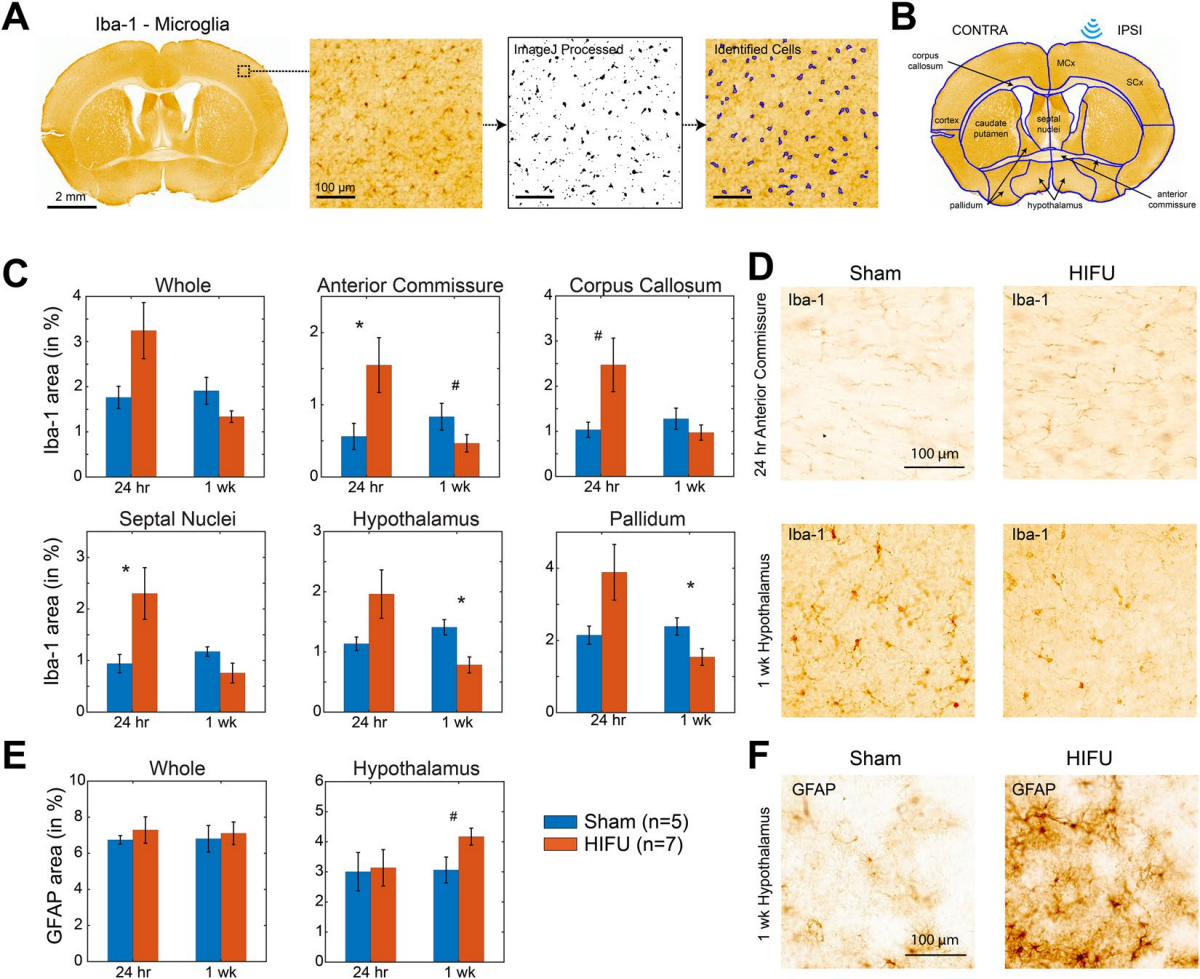
Astrocytes and microglia reactions to high-intensity focused ultrasound exposure. (**A**) Whole-mount coronal sections at ~0 Bregma stained with Iba1. Magnified images illustrate cell identification and quantification. (**B**) Same sections as in (A) with brain region and sonication location denotation. (**C**) shows the percentage of positive Iba-1 stained area to the total area of selected regions. Only regions show statistically meaningful difference between sham and HIFU exposed animals are included. (**D**) Representative Iba-1 images in anterior commissure at 24 hours post-treatment, and hypothalamus at 1 week post-treatment. **E**. show the percentage of positive GFAP stained area to the total area of selected regions. Only hypothalamus showed remarkably increase in astrocyte reactivity at 1 week post-treatment. **F**. Representative GFAP images in hypothalamus at 1 week post-treatment. (n = 5 in control groups, and n = 7 in HIFU groups at each time point. ^#^p < 0.1, *p < 0.05, Wilcoxon rank sum test. Please note that these findings did not show significance in post-hoc Benjamin and Hochberg false discovery rate correction, indicating glial reactions are mild).

To assess neuroinflammatory responses of the brain to HIFU exposure, ultrasound pulses were applied through a transducer and coupled nose-cone for a 20 second train of 40 sinusoidal pulses of 10 ms duration. The off time between pulses was 500 ms, resulting in a total exposure time of 20 seconds. The peak negative pressure was 13.5 ± 1.5 MPa measured in water (see Methods and Fig. 1B). Histological analysis of activated astrocyte and microglia were performed 24 hours and 1 week after ultrasound or sham treatment (n = 5 in control groups, and n = 7 in HIFU groups at each time point). We did not observe apparent localized glial reactions on sections from HIFU-exposed animals compared to those from sham controls (Fig. 2A). We therefore quantified the density of activated glial markers within each brain region found in a coronal section at Bregma 0 (Fig. 2B). Though no significant difference was identified when comparing the density across the whole slice between sham and ultrasound exposed groups (Wilcoxon rank sum test), cumulative distribution analysis across different brain regions revealed significant difference between the two groups (K-S test, p < 0.001). This suggests a region-specific effect. Rank sum test demonstrated that microglial markers were transiently increased at 24 hours post treatment in corpus callosum, anterior commissure, and septal nuclei (Fig. 2C,D). Additionally, at 1week post treatment, microglial activity was decreased in hypothalamus, anterior commissure, and pallidum. Reactive astrocytic markers increased in the hypothalamus at 1week post treatment (Fig. 2E,F), but not at 24 hours post treatment. However, these findings did not show significance in post-hoc Benjamin and Hochberg false discovery rate correction, indicating glial reactions are mild. No significant difference was detected between brain structures ipsilateral and contralateral to the ultrasound exposure for either marker.

These results suggest that the ultrasound pulse train caused mild regional neuroinflammatory responses extending beyond the theoretical primary focal site. This finding, while surprising, may be explained by constructive interference arising from reflection of waves within the cranium. In addition, the locations with significant glial reactivity are consistent with previous reports that white matter tracts and hypothalamus have a higher susceptibility to brain injury^20–24^.

### HIFU exposure impaired locomotor ability and exploratory behaviors in mice

Neurologic deficits related to brain injury include locomotor, cognitive and behavior symptoms such as reduced coordination and balance, and anxiety^25,26^. To assess the animals’ locomotor and exploratory behaviors, we use the rotarod and open field test (OFT), respectively.

**Figure 3:**
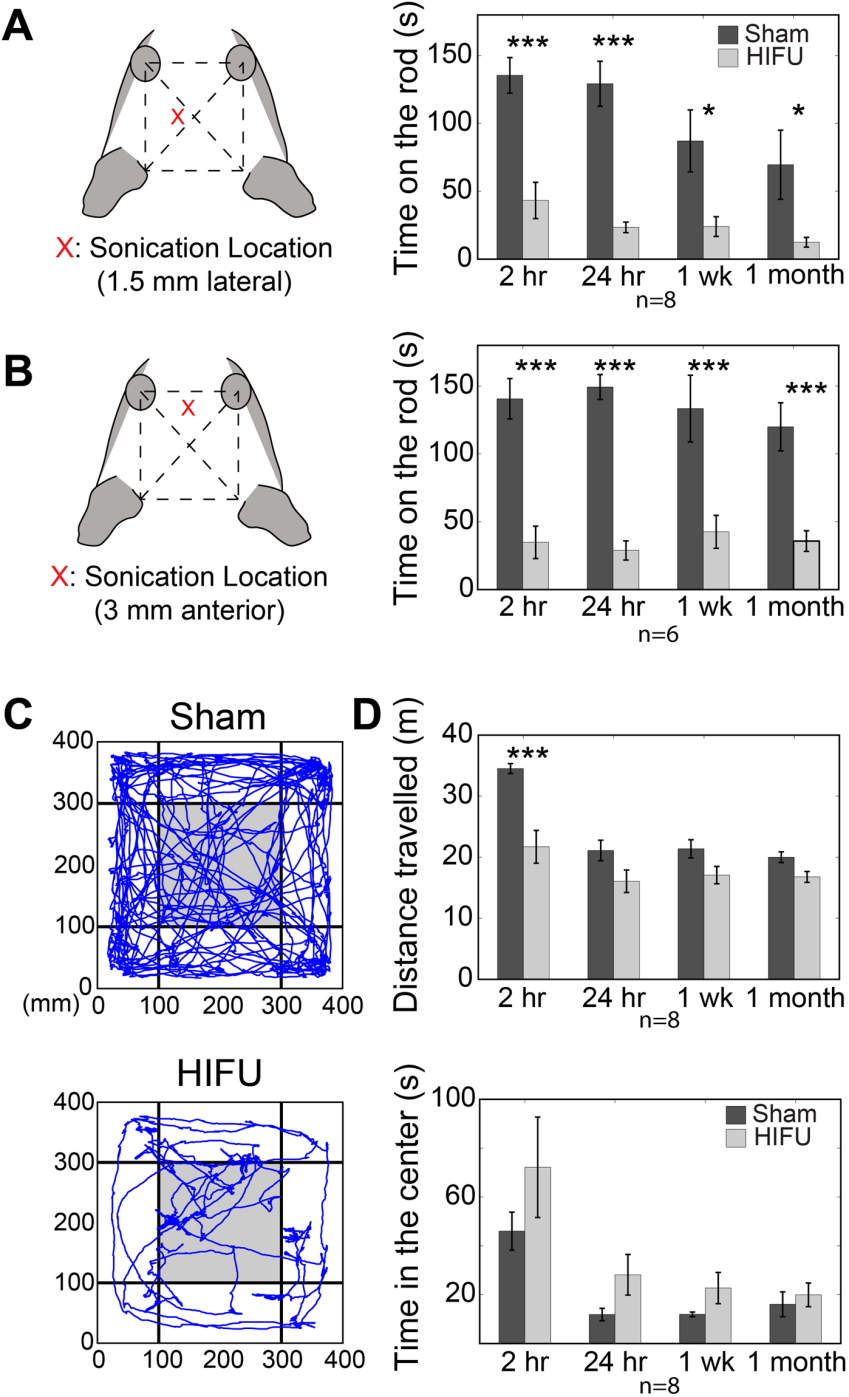
Behavioral deficits after focused-ultrasound exposure in mice. (**A**) HIFU exposure at approximately the motor cortex significantly reduced the latency that a mouse stays on a rotating rod (32 rpm) compared with sham group from 2 hours to 1 month after treatments. (n = 8 in each group). (**B**) Focused ultrasound sonication at approximately frontal cortex area also impaired mice’s locomotor ability, manifested as reduced latency at the rotating rod. (n = 6 in each group). (**C**) Representative traveling traces of mice in the first 5 minutes of an OFT 2 hours after sham and HIFU treatments respectively. (**D**) Animals subjected to HIFU travelled remarkably less distance compared to those in the sham group at multiple time points post treatment, indicative of reduced spontaneous exploratory activity (top). Animals subjected to HIFU exposure stayed longer in the center of the arena, defined as the gray region in (**C**), compared with sham (bottom). (n = 8 in each group) (Data expressed as mean ± se, *p < 0.05, **p < 0.01, ***p < 0.001, Two-way mixed-effects model followed by Sidak’s multiple comparison test [GraphPad Prism]).

Prior to rotarod testing, HIFU pulses were applied in the left primary motor cortex area with the same location and parameters as for histology (Fig. 3A, n = 8 for HIFU and sham groups). Animals that were subjected to HIFU exposure remained on the rod for shorter amounts of time than sham animals in tests performed at acute timepoints 2 and 24 hours after treatment. These deficits persisted chronically through 1 month after treatment (Fig. 3A and Table 1). To exclude the possibility that the poor performance of animals exposed to HIFU was due to the direct injury of the motor cortex, a second cohort of animals received HIFU exposure at midline, 3 mm anterior to the prior treatment location, approximately over frontal cortex. Animals subjected to HIFU in this cohort also showed profound deficits in the time spent on the rotarod at 2 hours, 24 hours, 1 week and 1 month timepoints post-treatment (Fig. 3B and Table 1; n = 6 in each group). In addition, animals treated with HIFU demonstrated higher variability in rotarod performance, measured as a higher coefficient of variation (CV: the ratio of the standard deviation to the mean value) (Table 1).

**Table 1:** Rotarod and OFT behavioral Testing.

	2 hours Post					24 hours Post				
	Sham		HIFU		P value	Sham		HIFU		P value
	Mean ± SE	CV	Mean ± SE	CV		Mean ± SE	CV	Mean ± SE	CV	
Rotarod (Motor Cortex) (s) (n = 8)	135.50 ± 13.09	0.2732	43.25 ± 13.37	0.8744	0.0003	129.28 ± 16.60	0.3631	23.38 ± 3.86	0.4673	<0.0001
Rotarod (Frontal Cortex) (s) (n = 6)	140.60 ± 14.90	0.2596	34.73 ± 11.94	0.8423	<0.0001	149.27 ± 9.15	0.1502	28.77 ± 7.05	0.6006	<0.0001
OFT: Distance Travelled (m) (n = 8)	34.52 ± 0.82	0.0670	21.70 ± 2.69	0.3505	<0.0001	21.10 ± 1.68	0.2249	16.05 ± 1.85	0.1150	0.1053
OFT: Time in the Center (s) (n = 8)	45.99 ± 7.78	0.4795	72.14 ± 20.58	0.8067	0.1685	11.84 ± 2.53	0.6049	28.11 ± 8.33	0.8384	0.6053
OFT: Freezing Time (s) (n = 8)	18 ± 2.84	0.4464	76.13 ± 22.24	0.8265	0.0249	71.88 ± 13.09	0.5149	113.50 ± 19.35	0.4821	0.1741
	**1 week Post**					**1 month Post**				
	**Sham**		**HIFU**		**P value**	**Sham**		**HIFU**		**P value**
	**Mean ± SE**	**CV**	**Mean ± SE**	**CV**		**Mean ± SE**	**CV**	**Mean ± SE**	**CV**	
Rotarod (Motor Cortex) (s) (n = 8)	87.08 ± 22.88	0.7432	23.98 ± 7.26	0.8560	0.0205	72.58 ± 26.00	1.0131	12.40 ± 3.62	0.8247	0.0426
Rotarod (Frontal Cortex) (s) (n = 6)	133.37 ± 24.57	0.4514	42.52 ± 12.12	0.6985	0.0002	119.93 ± 17.74	0.3624	35.70 ± 7.65	0.5249	0.0006
OFT: Distance Travelled (m) (n = 8)	21.37 ± 1.48	0.1970	17.06 ± 1.41	0.0829	0.2129	19.99 ± 0.87	0.1244	16.77 ± 0.89	0.1500	0.4876
OFT: Time in the Center (s) (n = 8)	11.90 ± 0.94	0.2222	22.68 ± 6.41	0.7994	0.8722	16.05 ± 5.08	0.8942	19.89 ± 4.84	0.6888	0.9969
OFT: Freezing Time (s) (n = 8)	73.75 ± 10.12	0.3879	108.75 ± 17.74	0.4614	0.3226	83.38 ± 11.35	0.3850	112.75 ± 9.01	0.2260	0.4944

(Two-way mixed-effects model followed by Sidak’s multiple comparison test [GraphPad Prism]).

To determine the impact of HIFU on animals’ basic exploratory behavior, we performed OFT in a separate cohort of animals (n = 8 in both HIFU and sham groups). HIFU pulses were applied at approximately the left motor cortex area as described above. Distance travelled, time spent in the center of the arena (20 cm × 20 cm square region, shown as gray in Fig. 3C), as well as time spent freezing in the first 5 mins were quantified. At 2 hours post-treatment, animals exposed to HIFU traveled significantly shorter distances and froze longer compared to sham (Fig. 3D and Table 1). In addition, HIFU exposed animals stayed in the center of the arena notably longer than the sham animals, though the effect was not statistically significant (Fig. 3D and Table 1).

Taken together, the rotarod and OFT data suggest that exposure to the HIFU pulse train impaired animals’ coordination and balance, and may also have altered cognitive factors that contribute to exploratory behavior. These behavioral and cognitive changes are consistent with previous reports on deficits related to mild brain injury^27–30^.

### Impact of HIFU exposure on ECoG signals

To better understand the effect of HIFU on neural function, we sought to characterize any resultant neurological deficits by recording ECoG signals from the surface of motor cortex. Longitudinal ECoG signals were measured from the right motor cortex for approximately 7 months (1month baseline for the contralateral experiment and 6 months post-treatment) (Fig. 1A). ECoG signals were quantified by absolute and relative power spectral density (PSD), power ratio between multiple frequency bands, sample entropy, and coherence. To eliminate movement-related artifacts from the signal, analysis was confined to epochs when the animal was quietly resting, determined through automated analysis of the animal’s behavioral state from video (Cleversys Inc., VA) and confirmed by analysis of EMG-related high frequency activity in the ECoG signal (Fig. 1C).

#### Acute ECoG signal change after HIFU exposure

To investigate the acute effect of sonication on the ECoG signals, we compared quantitative ECoG parameters between sonicated and sham groups at 24 and 48 hours after treatment recorded both ipsilateral and contralateral to the HIFU treatment.

Acute ECoG power spectra contralateral to HIFU: To allow for the collection of pre-treatment ECoG signals, and to account for individual differences in baseline signals, we conducted µECoG recording from the hemisphere contralateral to injury (Fig. 1B). This method allowed us to apply ultrasound with the electrode already implanted on the contralateral side (n = 12 mice), or apply a sham treatment (n = 10 mice). Two animals had presumed damage to the electrodes, manifested as a lack of high-quality ECoG signals, and the presence of exceptionally high (>5-fold of the other animals) 100–300 Hz oscillations after the injury, and were excluded from data presented below. For some time points, the number of animals is less than 10 due to excessive movement artifacts during that specific recording session, likely from locomotion or grooming, and those recordings were excluded from analysis (see Methods for exclusion criteria).

**Figure 4:**
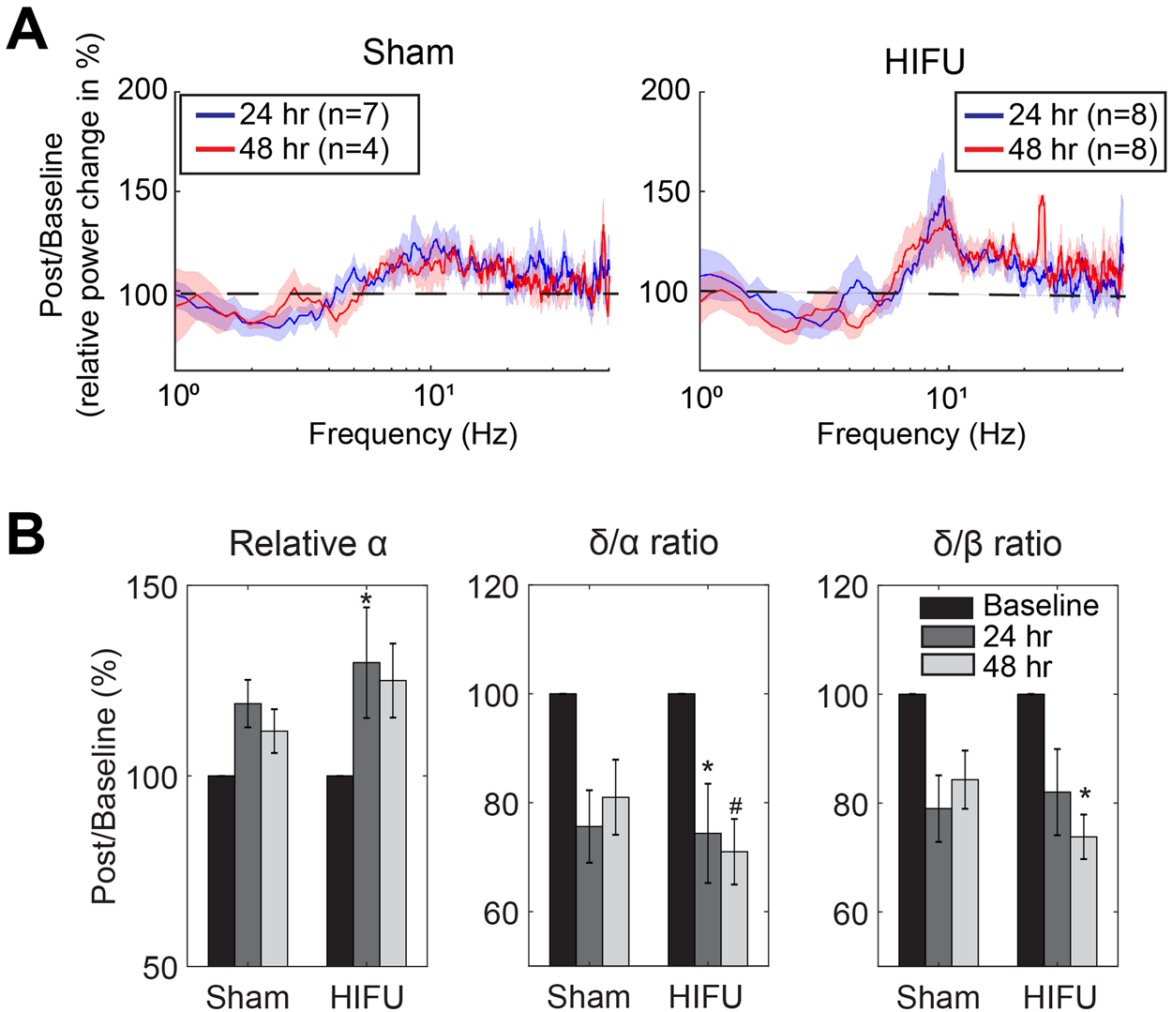
Acutely altered relative PSD compared to baseline on the contralateral hemisphere. **(A)** Relative power change calculated by dividing post-treatment relative PSD by that of the baseline. Baseline was the average of 4 pre-treament recordings. Shades represent SE. (**B**) Summary of the power spectral components that were significantly different from the baseline. (n = 7 and 4 in sham group at 24 and 48 hours respectively, n = 8 in injured group. #p < 0.1, *p < 0.05, Friedman and post-hoc Tukey tests.).

We first analyzed the temporal dynamics of power spectral changes following the HIFU procedure. At 24 hours post HIFU, both sham and HIFU exposed animals showed a trend towards reduction in low frequency oscillatory activity (relative δ; 1–4 Hz) and an increase alpha frequency activity (relative α; 8–13 Hz), leading to reductions in the δ/α and δ/β power ratios (Figs 4 and S1). However, these reductions were significant only in HIFU animals and persisted through 48 hours post treatment (Friedman test and post-hoc Tukey test). When comparing the HIFU animals directly to the sham animals, HIFU animals showed a higher δ/β ratio and lower relative β power than sham animals at 24 hours post injury (Fig. S2). Supplemental Fig. 1 summarizes the ECoG parameters that showed statistically meaningful changes following treatments (p < 0.1, n = 7 and 4 in sham group at 24 and 48 hours respectively, n = 8 in injured group, Friedman test).

To determine if initial common effects between the sham and HIFU groups were due to isoflurane anesthesia, we included a control cohort of animals that were only exposed to isoflurane for 30 mins after 4 weeks baseline data collection. Isoflurane exposure alone produces subtle effects in population synchrony, with a trend towards a reduction in the relative power at δ band and an increase at α and β bands, although these results were not significant in this cohort (n = 4, Fig. S3). This leads us to conclude that part, but not all, of the acute effects observed can be attributed to the effect of isoflurane.

These data suggest that while isoflurane and surgical procedures had an evident impact on local cortical activity for 24 hours, sonicated animals displayed additional deficits that appeared to evolve through 48 hours post-injury. The reduced low-frequency activity (δ) and increased higher frequency activity are likely due to effects of ultrasound modulation, and are partially obscured at earlier timepoints due to the after-effects from the surgical protocol.

Acute ECoG PSD ipsilateral to HIFU: Although performing ECoG recording contralateral to the side of HIFU allowed us to collect baseline activity, it did not let us directly investigate the changes to circuit function proximal to ultrasonication. Therefore, to investigate how HIFU affects local circuit function, we performed a second ECoG cohort that had recording electrodes positioned directly above the area of sonication. To avoid ultrasound wave damaging the µECoG array, we sonicated the right motor cortex through the skull immediately prior to craniotomy and implantation of the electrode array (Fig. 1A, indicated in purple).

**Figure 5:**
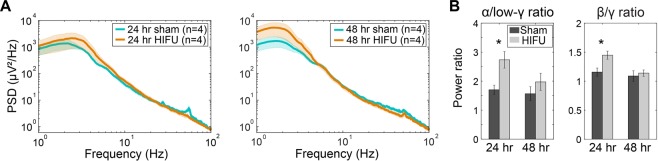
Acutely altered PSD after injury on the ipsilateral hemisphere to HIFU. (**A**) PSD at 24 and 48 hours post treatment. Shaded areas represent SE. (**B**) Mice exposed to HIFU had significantly higher α/low-γ and β/γ ratios at 24 hour post injury compared to sham animals. (n = 4 in each group at each time point. *p < 0.05, Two-way mixed-effects model followed by Sidak’s multiple comparison test [GraphPad Prism]).

Figure 5A shows the PSD of sham and injured mice at 24 and 48 hours post treatment from ipsilateral to the sonication. In contrast to changes in activity of the contralateral hemisphere, the ipsilateral hemisphere of HIFU-treated animals demonstrated higher δ oscillations, but lower γ frequency oscillations, compared to sham animals (Figs 5A and S4), though these did not reach statistical significance. Statistical analysis revealed significantly higher α/low-γ (low-γ is oscillations at frequency between 30 and 56 Hz) and β/γ ratios in injured animals at 24 hours post injury (Figs 5B and S4) (n = 4 in each group at each time point).

Temporal dynamics of acute injury: One limitation of the previous set of analyses of contralateral ECoG signals is reliance on baseline measures, which are rarely present in human clinical detection of brain injury, hindering the translational possibility for those measures. Moreover, this prevents direct comparison between contralateral and ipsilateral results, as the ipsilateral paradigm does not allow for the collection of baseline data.

**Figure 6:**
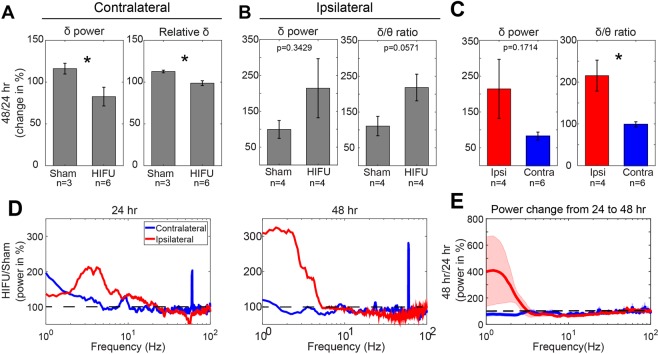
Differential PSD changes between contralateral and ipsilateral hemispheres to HIFU acutely after the injury. (**A**) On the contralateral side of the brain HIFU, sham and injured mice showed a divergent trend of change in absolute and relative δ power from 24 to 48 hours post treatment. (n = 3 in contralateral sham group, n = 6 in contralateral HIFU group, and n = 4 in ipsilateral HIFU and sham groups). (**B**) On the ipsilateral side of the brain to HIFU, injured animals showed increases in δ power and δ/ɵ ratio from 24 to 48 hours post treatment compared to sham control. (n = 4 in each group). (**C**) Differences in the change of δ power and δ/θ ratio from 24 to 48 hours post injury between ipsi- and contra-lateral sides of HIFU. (n = 6 in contralateral group, and n = 4 in ipsilateral group). (**D)** Alterations of PSD at ipsi- and contra-lateral sides to the injury at 24 and 48 hours post HIFU exposure. It was calculated by dividing the mean PSD of the injured animals by that of the sham animals. (**E**) Change of PSD from 24 to 48 hours post treatment on ipsi- and contra-lateral sides of the injury. Note the increase in δ frequency band in mice with injury at the ipsilateral side of the ECoG recording, whereas, decrease in animals with injury at the contralateral side of the recording. Shaded areas represent SE. (*p < 0.05, Wilcoxon rank sum test).

To address this, we performed a within-animal analysis of the changes in spectral content between 24 and 48 hours post treatment in both contralateral and ipsilateral injury animals. This analysis largely mitigates the inter-subject baseline variability, and provides insight into the temporal evolution of the response to HIFU. Between the 24 and 48 hour recovery period, contralateral recordings in HIFU animals demonstrated decreases in absolute and relative δ (Fig. 4A). In contrast, sham animals showed increases across these measures, demonstrating a recovery towards baseline levels (Fig. 6A; sham vs. HIFU; δ: 116.09 ± 6.38% vs. 82.59 ± 11.18%, p < 0.05; relative δ: 112.64 ± 1.60% vs. 98.71 ± 2.87%, p < 0.05. Wilcoxon rank sum test, n = 3 in sham, and n = 6 in injured group).

Measurements ipsilateral to HIFU show the opposite trend, with a large increase in the power at δ frequency in HIFU treated animals between the 24 to 48 hour timepoints. In addition, ipsilateral measurements show a slight reduction in the power at ɵ, α, and β bands (Fig. 6E), resulting in apparent increase in δ/ɵ ratio, compared to sham animals (Fig. 6B) (110.71 ± 27.17% vs. 218.40 ± 37.29%, p = 0.0571, n = 4 in each group). Together, these results suggest that the ipsilateral and contralateral hemisphere have distinct temporal dynamics in the initial days post-injury.

Differential acute PSD changes between contralateral and ipsilateral hemispheres: In addition to the above changes we observed in ipsilateral or contralateral sides to the injury, we identified disparity in the acute PSD change between contralateral and ipsilateral hemisphere to the injury (Fig. 6C–E), in both cases normalized to the sham condition to control for surgical differences between the groups. At 24 hours post injury, both hemispheres showed an increase in δ oscillations compared to sham, which was more pronounced at the ipsilateral side. Ipsilateral δ power continued to increase within 48 hours post injury, whereas contralateral side began to decrease (Fig. 6D). These divergences are associated with significant difference in the change of δ/θ ratio between two hemispheres from 24 to 48 hours post-injury (Fig. 6C) (215.48 ± 36.97%, n = 4, in ipsilateral group; 98.83 ± 6.3%, n = 6, in contralateral group), pointing again to the divergent evolution of spectral changes between the two hemispheres.

Taken together, these data demonstrate that exposure to the ultrasound caused acute alterations in PSD of surface brain electrical signals that persisted through 48 hours post treatment, suggesting PSDs have the potential to be used to identify a brain functional abnormality within 48 hours after injury.

No acute change in the local coherence after exposure: To determine the impact of injury on the functional coupling between different recording sites, we computed coherence between all pairwise electrode combinations. As expected, a negative correlation was present between the coherence coefficient and the distance between electrodes. Likewise, correlation decreased with increasing oscillation frequency, as previously reported^31^. However, we did not observe differences in either coherence or change from the baseline between sham and injured animals at 24 and 48 hours post treatment on both contralateral and ipsilateral sides of injury. In addition, no obvious difference was detected in the recovery/progression of coherence from 24 to 48 hours post treatment between sham and injured animals. The lack of changes in coherence may be due to the small area of the brain that the µECoG array can cover (2 × 2 mm).

Acute sample entropy after exposure: We quantified the sample entropy of surface LFP signals to capture nonlinear temporal dynamics of brain activities, which reflect the complexity of the signal and indicate the number of signal sources (i.e. the more irregularly that neurons are firing, the higher the entropy). On the contralateral side of the injury, we observed a slight, but non-significant, reduction in entropy at 24 hours post injury, which recovered toward pre-treatment values from 24 to 48 hours after HIFU exposure. On the ipsilateral side of the injury, the injured animals also showed lower entropy at 24 and 48 hours compared to those subjected to sham treatment (Fig. S4). In contrast to the contralateral side of the injury, sample entropy continued decreasing from 24 to 48 hours ipsilaterally with a 22.36 ± 15.07% reduction, whereas sham animals had a 20.80 ± 23.74% increase (p = 0.34), pointing again to a slower resolution of circuit disruption on the same hemisphere (ipsilateral) as HIFU exposure. Though not statistically significant, the sample entropy analysis revealed an acute trend towards reduction in the irregularity/complexity of brain surface electrical signals, suggesting a decrease in the number of signal sources after HIFU exposure.

#### Long term ECoG signal change after HIFU exposure

To understand the significance of ultrasonic injury over time, we obtained longitudinal recordings from all implanted cohorts for 6 months. ECoG signals were recorded weekly or biweekly from the first week post treatment. The temporal dynamics of ECoG signals were gradual, allowing us to group the data into three time windows, 1–5 weeks, 6–12 weeks, and 14–24 weeks post treatment. The selection of the three chronic time windows is based on visual observation of the trend of ECoG parameter changes over time. We first quantified the ECoG spectral power for each recording, then averaged the quantified parameters within each time window for each animal.

**Figure 7:**
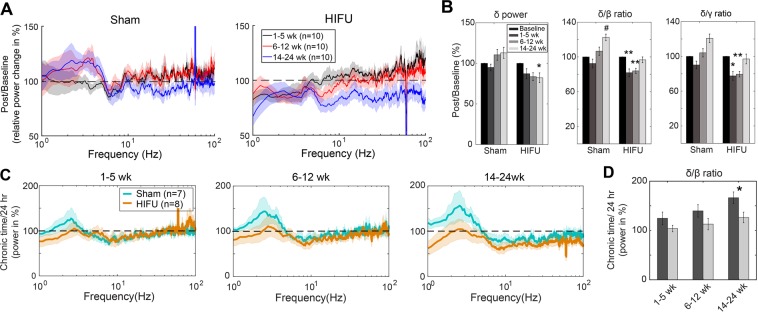
Chronically altered PSD on the contralateral hemisphere. (**A**) Relative PSD change compared to the baseline, calculated by dividing post-treatment relative PSD by that of the baseline. Baseline was the average of 4 pre-treament recordings. (^#^p < 0.1, ^*^p < 0.05, ^**^p < 0.01, Friedman test and post-hoc Tukey test, n = 10 in each group at each time point). (**B**) Summary of the power spectral components that were significantly different from the baseline. (**C**) Change of PSD from 24 hour to chronic time windows post treatment. Note the gradual increase in low frequency oscillation and decrease in high frequency oscillation in sham animals, whereas relatively stable low frequency oscillations in injured animals. (n = 7 in sham group, and n = 8 in HIFU group). (**D**) Sham and injured animals showed significantly different trend of change in δ/β ratio from 24 hours to 14–24 weeks post treatment. (*p < 0.05, Two-way mixed-effects model followed by Sidak’s multiple comparison test [GraphPad Prism]) (Shaded areas in (**A**,**C**) represent SE.)

Temporal dynamics of power spectra on the contralateral side of the injury compared to baseline: Compared to the pre-HIFU baseline, HIFU animals demonstrated a significant reduction in δ for 6 months following injury (Fig. 7B). A simultaneous increase in higher frequency activity results in reduced δ/β ratio, and δ/γ ratio that persists through 12 weeks post injury and then resolves (Fig. 7) (n = 10 in each group at each time point). To gain additional insight into post-injury dynamics, and create measurements not reliant on baseline values, we normalized power to the value at 24 hours post injury, and evaluated all subsequent timepoints. HIFU and sham animals have inverse directions of change at δ frequencies, with HIFU animals showing evolving decreases in power and sham animals showing increases. Both groups show reductions in higher frequencies, but at a greater magnitude in HIFU animals. The cumulative effect of these changes manifests in a significant difference between the δ/β ratio at the final time window (14–24 weeks; 166.41 ± 11.71% vs. 126.43 ± 10.72%, p = 0.042, n = 7 in sham and n = 8 in HIFU group) (Fig. 7D).

**Figure 8:**
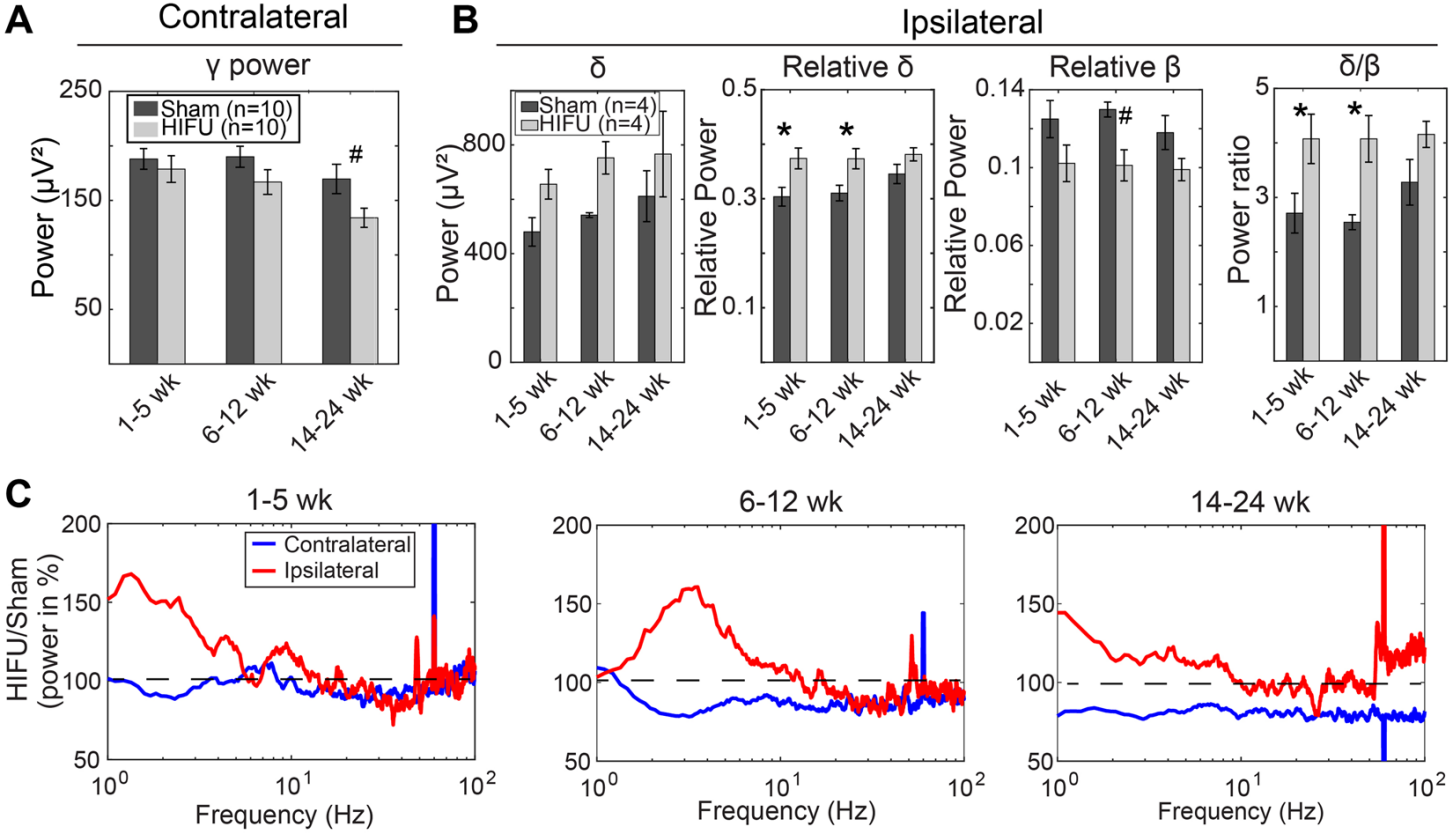
Differential chronic PSD changes between contralateral and ipsilateral hemispheres. (**A**) On the contralateral hemisphere to the treatment, injured mice had significantly lower γ power at 14–24 weeks post HIFU compared to sham animals. (n = 10 in each contralateral group at each time point). (**B**) On the ipsilateral hemisphere, injured mice had significantly higher relative δ and higher δ/β ratio at 1–5 weeks, and 6–12 weeks after HIFU, and lower relative β at 6–12 weeks post injury. (n = 4 in each ipsilateral group at each time point). (**C**) Difference in the PSD between ipsilateral and contralateral groups at chronic time windows. It was calculated by dividing the mean PSD of the injured animals by that of the sham animals at the same time window. Note the higher low frequency oscillations on the ipsilateral side to the injury, whereas lower on the contralateral side. (^#^p < 0.1, *p < 0.05, Two-way mixed-effects model followed by Sidak’s multiple comparison test [GraphPad Prism]).

Temporal dynamics of HIFU treated animals compared to sham controls: When directly comparing PSD of HIFU and sham groups in the contralateral experiment, HIFU animals demonstrated lower broad spectrum (1–100 Hz) power, with increasing difference from sham animals over time (Figs 7A and 8C). Strikingly, while both groups showed a gradual reduction in γ frequency activity, the reduction was larger in HIFU animals, reaching significance at the latest timepoint (14–24 weeks) (Fig. 8A). Taken together, the persistent reduction in δ activity, and the accelerated dynamics of the reduction in γ frequency activity in the HIFU animals suggest an evolving response to injury that lasts for 6 months after the insult.

Chronic measurement of the hemisphere ipsilateral to HIFU revealed increased low frequency oscillations in injured animals at 1–5, 6–12, and 14–24 weeks post treatment (Fig. 8B,C). Statistical analysis revealed significantly higher relative δ power and lower relative β power at 1–5 weeks and 6–12 weeks time windows (n = 4 in each group at each time point) (Fig. 8B). These changes in individual frequency bands caused significantly higher δ/β ratio that persists through 12 weeks post injury (Fig. 8B).

Differential chronic PSD changes between contralateral and ipsilateral hemispheres: Overall, the difference in power during chronic timepoints between injured and sham animals was greatest on the ipsilateral hemisphere, particularly for low frequency oscillations (Fig. 8C). In addition, spectral changes on the ipsilateral hemisphere were of opposite valence to those of the contralateral hemisphere. These results are in direct contrast to the results from the contralateral hemisphere (Fig. 7C), where low frequency oscillations remain stable at chronic timepoints, suggesting a lateralization of injury not revealed by histology. Moreover, the dynamics of these spectral changes were inverted; ipsilateral δ oscillations became more similar to sham animals, while contralateral δ oscillations trended away from the sham animals (Fig. 8C). This may be explained due to dynamics within the sham animals, such as a gradual increase in δ activity, possibly due to aging^32^, which masks the effects on the injured hemisphere (Fig. 8B,C).

Taken together, the data reveal complex and lasting alterations in inter- and intra-hemispheric circuit function post HIFU, indicative of a persistent brain injury.

## Discussion

This study provides both behavioral and electrophysiological evidence of long-term neurological deficits arising from exposure to a train of intense ultrasound pulses, despite a lack of a focal neuroinflammatory response. We observed pronounced locomotor deficits, mild diffuse neuroinflammatory responses, and altered brain surface electrical signals at both acute and chronic timescales following HIFU. Low frequency (δ) and high frequency (β, γ) oscillations were all altered at acute and chronic timescales by the application of ultrasound. ECoG changes on the hemisphere ipsilateral to HIFU exposure were of greater magnitude than the contralateral hemisphere, and persisted up to three months post-treatment. Between 24 and 48 hours post treatment, δ power and δ/θ ratio decreased on the side contralateral to injury, but were markedly increased on the ipsilateral cortex. Ipsilateral increase in δ power and decrease in higher frequency activity was maintained for 12 weeks post injury. Interestingly, the contralateral hemisphere developed an alteration in high frequency activity at 12 weeks that persisted through 6 months post treatment. These results suggest the possibilities for long-lasting neurological damage following exposure to trains of high-intensity ultrasound pulses even in the absence of focal lesions.

It is important to characterize the ultrasound field giving rise to the observed deficits. The peak negative pressure measured in water was 13.5 ± 1.5 MPa. The peak positive pressure was measured to be 65 ± 10 MPa. The pulse train was thus comprised of pulses that had undergone considerable steepening. The pressure amplitudes in the mouse brain will be somewhat lower owing to absorption and defocusing by the skull. A recent study in rodents reported a pressure transmission rate of 82% at a frequency of 1.5 MHz^33^. Using the 82% transmission rate, the *in-situ* peak negative pressures in the present study would be approximately 11 MPa. Such pressure levels are in the range where cavitation is possible. In a summary report on cavitation effects, cavitational thresholds in the brain were identified as 12.0 MPa at 0.66 MHz (sheep model), 10.4 MPa at 0.94 MHz (rabbit), and 13.6 MPa at 1.72 MHz (rabbit)^34^. In the present study involving the mouse brain, attenuation along the short propagation path is minimal, and constructive interference following reflection of the skull base may have resulted in pressures higher than 11 MPa. Despite the potential for cavitation, we did not observe signs of cavitation, such as pitting or hemorrhage, in gross observation of brain tissues. Prussian blue staining in slices at Bregma 0 demonstrated significantly increased number of iron spot at 24 hours post treatment, however, the total area of positive stain was not significantly different between HIFU and sham groups (Fig. S5). It is reasonable to conclude that cavitational effect of the operating regime for our sonications was minimal.

In addition to cavitation, thermal damage must be considered when pressure pulses of the magnitude used in this study are employed. A full thermal analysis is beyond the scope of this article, due to the need to properly account for attenuation of the shocked waveform and heat transfer in a highly perfused organ. We refer instead to the empirical observations that the apparent denaturization characteristic of thermal damage was not visible in the histological analyses, which are expected to be shown as focused increased glial reactions in response to tissue necrosis or ischemia. In addition, *in-situ* temperature measurements next to the focal region (described in the Methods section) showed temperature rises on the order of 3 degrees Celsius.

Rather than cavitational or thermal effects, the neurological deficits observed in this study may originate from the radiation force exerted by the beam on the nerves. Gradients in the radiation force, produced by radial pressure variations in the ultrasound beam, or by differential absorption by neighboring tissues, can generate localized shear stresses that cause mild injury. Sonication in an earthworm model showed that reduced nerve functionality correlated with the cumulative radiation force impulse^35^, given by

$$CRFI=\frac{\alpha d{p}_{RMS}^{2}{t}_{pulse}N}{{\rho }_{0}{c}_{0}^{2}},$$

where *α* is the absorption coefficient, *d* the ultrasound beam width, *p*_*RMS*_ the root-mean-square pressure, *t*_*pulse*_ the on time of the pulse, *N* the number of pulses, *ρ*_0_ the density of the medium, and *c*_0_ the speed of sound of the medium. The CRFI is a dose metric, involving both pressure level and exposure duration, analogous to the thermal dose used to predict damage due to heating. Whether the CRFI is useful for correlating the deficits observed in the present study requires further study with different parameter combinations. For future comparison, we provide here the CRFI value using the exposure conditions employed in our investigation. The RMS pressure of the waveform described above was approximately 19 MPa. Using an absorption coefficient in the brain of 0.024 cm^−1^^36^, a pulse diameter of 1 mm, a pulse temporal width of 10 ms, N = 40 pulses, *ρ*_0_ = 1000 kg/m^3^, and *c*_0_ = 1500 m/sec, we obtain a CRFI of roughly 150 Pa sec. Should CRFI prove to be useful in predicting the neural deficits revealed in the present study, the 150 Pa sec value would be useful in predicting deficits for procedures performed at much lower pressures but for considerably longer periods of time, such as acoustic neuromodulation. Similarly, in histotripsy procedures, the pressure magnitudes exceed those of this study, and the 150 Pa sec CRFI value could provide a useful safety metric as brain histotripsy approaches clinical application. We note that the 150 Pa sec CRFI estimate is not conservative in the mouse model, in the sense that constructive interference within the mouse skull could lead to higher pressure values (by a factor of 2 in the most extreme case) in certain locations, and the CRFI would subsequently be higher (potentially up to 300 Pa sec). This increase in CRFI due to reflection would be considerably less in a human brain, where the energy loss accruing along the propagation path is much larger and the reflections smaller.

Histological assessment is widely used to study the safety of FUS. Though structural characterization of brain tissue following HIFU is not a focus of this study, it is important to understand the correlation between functional changes and the level of tissue responses. Within the immune-privileged environment of brain, glial cells are the primary drivers of neuroinflammation in response to brain injury^37–40^. Therefore, we focused our analysis on microglial (Iba1) and astrocyte (GFAP) reactions, which are the common responders to various types of tissue damages, i.e. blood-brain barrier opening and neuronal necrosis. For instance, recent work shows that GFAP has strong predictive power for brain injury following trauma, and is strongly associated with neurodegenerative disease and stroke^41^. With the 20 second dose consisting of 40 pulses, we did not observe reactive microglia or astrocyte reactivity in the predicted ultrasound focus, nor did we find neuroinflammation lateralized to the side of sonication, indicating focused damage. Instead, we identified a more diffuse, mild and bilateral pattern of microglia and astrocyte activation in select brain regions including white matter tracts, hypothalamus, septal nuclei and pallidum. The histological result is in line with previous reports that structures lining ventricles, including corpus callosum, hypothalamus, and thalamus, appear to have higher susceptibility to impact, particularly blast^20,42–44^. One limitation of these studies is the inherent semi-quantitative nature of immunohistochemistry, which may limit the sensitivity of our analysis. In addition, the diffuse neuroinflammatory response may not translate to humans, since only a small fraction of the human brain volume would be exposed to significant ultrasound energy. For the mouse, the beam width (especially in the axial direction) covers a significant fraction of the brain.

Although the neuroinflammatory response to a HIFU-pulse train of this intensity seems to be relatively mild, the mice showed consistently impaired locomotor ability that persists for at least a month post-exposure. As measured by both time spent on the rotarod and distance traveled in the open field arena, sonicated animals showed impaired locomotor capabilities compared to sham animals throughout the 4 weeks after injury, which may contribute to the noticeable increased dwell time in the center of the open field arena. Behavioral deficits were similar in phenotype, magnitude and duration regardless of the precise location of HIFU application (located over motor cortex vs. over prefrontal cortex). This is consistent with the diffuse, and non-lateralized nature of the neuroinflammatory response. This suggests that the effects of the exposure were not spatially localized to a focal region below the transducer, but instead distributed, both in terms of inflammatory patterns and effects on cognition. It is possible that the small path length of the ultrasound in the mouse skull leads to reflections over a range of directions, and consequently a diffuse spatial distribution of damage. Another explanation may be the more prominent damage in the white matter tract as evidenced in the microglial reaction, which can possibly result in neuronal degeneration in afferent and efferent regions. Indeed, it appears that the density of NeuN staining is slightly lower in sonicated animals than sham control 7 months after treatments (Fig. S5, statistics was not performed in this dataset due to limited number of animals, n = 2 in each group). However, microscale damage that is less robustly detected by microglial and astrocyte responses, such as axonal degeneration and microbleeds, cannot be ruled out as a contributing factor. Another possible explanation is that the effect of ultrasound on the brain may be through an indirect auditory pathway^45,46^, which leads to damage in the vestibular system. However, this requires further investigation, and may not fully explain the reduced activity in open field test.

To understand the impact of ultrasonic application on brain function, we used longitudinal ECoG recording to perform quantitative electrophysiological measurements of local neural population activity from the surface of the brain. Previous reports have described the alterations of quantitative electrophysiology following brain injury, including PSD and coherence analysis at limited post-injury time points (summarized in^47–52^). We collected surface brain activity with implantable electrodes longitudinally from 4 weeks prior to 6 months following ultrasonic treatment applied in a highly controlled dose and spatial location. Moreover, we performed analysis on the brain signals by quantifying multiple ECoG parameters from 1 to 100 Hz, including PSD, coherence, and entropy, which can provide a comprehensive picture of brain function.

We found an increase in δ power and a reduction in β and γ ipsilateral to the injury from 24 hours to up to 3 months post injury. These findings are consistent with previous studies in both human TBI patients and rodent injury models, which reported an increase in low frequency brain oscillations and decrease in high frequency oscillations at sub-acute and chronic phases^53–59^. This lends support to the idea that the HIFU exposure could cause an injury with similar electrophysiological characteristics to a TBI. Increased δ oscillations in the resting, awake brain may be associated with increased sleep needs following injury. δ oscillations are most prominent during non-rapid eye movement (NREM) sleep in healthy subjects, and increase in the cortex in awake subjects following sleep deprivation^60,61^. Patients with TBI have increased sleep needs for a long time after the injury^62^. Mice subjected to TBI also have more NREM sleep compared to sham control^59^. In contrast, high frequency oscillations (β and γ) are often associated with cognitive tasks, i.e. attention, memory, and sensory perception^63–67^. Indeed in TBI patients, it was found that decreased β and increased δ oscillations are correlated with reduced cognitive function^56^.

Interestingly, in contrast to increased δ on the ipsilateral hemisphere of the injury, we observed a reduction of δ power on the contralateral side to the injury, of a smaller magnitude. Multiple studies of brain oscillations following TBI have shown that changes in δ power can be brain region-specific. A recent MEG study indicates a spatial distinction in the change of spectral content following TBI, with parietal regions showing reduced δ power while other brain areas show increases^68^. Another study on chronic blast TBI patients also show some brain regions, including prefrontal and right temporal cortices, have more prominent δ power increase^55^. Considering that we performed ECoG recording from the same location in both contralateral and ipsilateral experiments, we think the difference in post-injury δ power change may be correlated with primary injury site. Additionally, it may be possible that the HIFU application immediately prior to craniotomy for the ipsilateral condition may have increased the susceptibility to surgery-related brain injury. Changes of opposite valence between the hemispheres may indicate reduced inter-hemisphere functional connectivity caused by the damage to white matter tracts revealed by our histological results. Indeed, multiple studies have reported reduced global coherence or inter-hemisphere function connectivity following TBI^53,69^, though it can be network specific^70^.

Another interesting finding of this study is the effect of aging on ECoG signals. In both contralateral and ipsilateral experiments, sham animals show consistent increase in low frequency oscillations (δ), and decrease in high frequency oscillations (β, and γ) at long-term time points. This is consistent with recent report that old mice have increased PSD at about 3 Hz, while decreased between 10 to 15 Hz during waking status^32^. However, this makes the interpretation of post-HIFU ECoG signal changes complicated. The ipsilateral hemisphere showed a resolution of all differences between HIFU and sham animals by 12 weeks post-injury. This can be indicative of a complete recovery process, or suggestive of an early onset of aging. In parallel, contralateral high frequency oscillations become suppressed relative to sham animals, reaching significance after 12 weeks post sonication, suggesting a compensatory process evolving as the ipsilateral hemisphere recovers.

In addition, entropy, which reflects the amount of randomness contained in EEG signals, has been used in the monitoring of depth of hypnosis^71^ and may indicate brain injury^72,73^. A correlation between reduced entropy and reduced consciousness has been observed^73^. Consistent with these reports, we observed a non-significant but apparent acute reduction in sample entropy within 48 hours post sonication. The level of reduction is greater ipsilateral to injury compared to the contralateral hemisphere, but recovers over several weeks. Though physiological implications of EEG sample entropy remain to be fully unraveled, these results suggest entropy may serve as a useful factor in discrimination between control and subjects exposed to stress waves.

The similarities in functional and structural manifestations between injury produced in this study and blast brain injury have implications for brain injury modeling. The ultrasound-based mTBI research tool developed by McCabe *et al*., and modified for this investigation, has the potential for providing a mechanism to induce mTBI in animal models that is much more controllable, safe, and inexpensive than shock tubes or actual blasts. An ultrasound-based model is valuable for executing the high-throughput experiments necessary for studying mechanisms of mTBI, or evaluating new neurotherapeutics. However, validation of the HIFU-based model by electrophysiological means or behavioral methods had not been previously performed. The results of the present study reveal that the ultrasound-based model can generate noticeable, long-term behavioral deficits and electrophysiological changes. These findings are consistent with symptoms demonstrated by warfighters diagnosed with mild blast injury - medical images may show no apparent structural abnormalities, but the individuals possess long-term cognitive, proprioceptive, and sensory impairments^74–76^.

Though our study clearly demonstrates a brain injury induced by HIFU exposure, the disparity between minimal glial reaction and apparent behavioral deficit suggests further investigation is needed to identify the exact tissue damage mechanism, i.e. neuronal cell death, axonal damage, and blood brain barrier breakage or vestibular system damage. In addition, there are several confounding factors in the interpretation of our surface brain electrical activity data. One is the implantation of µECoG array. The implantation procedure involves craniotomy, which can produce a certain level of brain injury itself. To control for surgical variables, we conducted age-matched, implanted surgical control cohorts that were exposed to sham ultrasound procedures. Additionally, contralateral HIFU animals were allowed 4 weeks to recover from the craniotomy and subsequent µECoG array implantation. This should be sufficient time for acute inflammation to stabilize^77,78^. A second confound is the use of isoflurane in the HIFU procedure. As demonstrated in the Supplemental Fig. 3., a short duration of isoflurane exposure induces a non-significant alteration of ECoG signals for up to 48 hours. One additional limitation is that data from the ipsilateral and contralateral hemispheres were collected in different cohorts. Future work can include recordings from dual-hemisphere µECoG arrays, or through the use of dual hemisphere epidermal arrays to eliminate surgical confounds^10^.

In summary, our study indicates that exposure to an intense HIFU pulse-train can produce lasting behavior deficits and diffuse neuroinflammation concentrated in white matter regions. The deficits do not appear to originate from cavitation or heating. In addition, a single application can elicit short term and long term alterations in brain oscillatory activity for periods up to 6 months. The methods presented for producing neurological deficits and monitoring them via behavioral and electrophysiological means can be used to further define the safety envelope for therapeutic ultrasound, and to produce mTBI for purposes of improving diagnosis and treatment.

## Methods

### Animal use procedures

All animal use procedures in this study were approved by the FDA White Oak Institutional Animal Care and Use Committee and comply with the National Institutes of Health Guide for the Care and Use of Laboratory Animals. Adult male C57BL/6 J mice aged 2–12 month were used. For focused ultrasound exposure procedures, animals were anesthetized with 4% induction dose of isoflurane (Henry Schein), and then positioned in a stereotaxic apparatus (David Kopf Instruments). Animals were maintained under anesthesia with 1.5% isoflurane, body temperature was maintained at ~ 37 °C with a thermostat-controlled heating plate (Model TC-1000, CWE Inc.) and respiration rate was monitored and maintained at ~ 100 breaths/min during the procedure. To prevent reflection of ultrasound waves, the fur on the scalp was first clipped, and fully removed through application of a removal lotion (Nair, Carter Products), which was applied to the skin and then cleaned with saline.

For electrocorticography (ECoG) signal acquisition, 16-channel µECoG recording arrays sized 2 mm by 2 mm (NeuroNexus Technology) were implanted on the surface of the right motor cortex. Vital signs of the animals were monitored and maintained as described above throughout the implantation surgery. After retraction of the scalp, the skull was cleaned with saline and then a small craniotomy (2.5 mm × 2.5 mm) was drilled over the right motor cortex area (coordinates relative to Bregma in mm: AP + 1.5 to −1, L 0.5 to 3) using a high speed dental drill (Osada, 0.25 mm drill bit). µECoG arrays were placed on the surface of the cortex above the dura and secured by tacking two corners of the array underneath the skull. The array was covered with a silicone (Kwik-cast, WPI), and the wire secured with dental cement (C&B Metabond). A burr hole was drilled immediately anterior to the lambdoid suture for the anchoring of a custom-made ground pin as common reference (see Fig. 1B for location). Percutaneous connectors were adhered to the skull with dental cement. The free edge of the scalp was sealed with tissue adhesive (Gluture, MWI Veterinary).

### High-intensity focused ultrasound (HIFU) exposure

The HIFU system was composed of a power amplifier (A300, Electronics & Innovation), a function generator (AFG 3102 C, Tektronix), a 1.1 MHz ring-geometry ultrasound transducer (H102 model, Sonic Concepts) and a coupling water-filled nosecone with a 5 mm diameter orifice. Mice were anesthetized with isoflurane, and positioned in stereotaxic apparatus; and vital signs were monitored and maintained as aforementioned. The transducer and coupled nose-cone was placed ~2 mm above the skull (for implanted animals) or scalp (for histological and behavioral animals) of the anesthetized mice, and the amplifier was turned on for experimental animals or left off for sham controls. For both experimental and control groups, the entire procedure took 15 to 30 mins. The ultrasound dose consisted of a train of 40 sinusoidal pulses, each of duration of 10 ms followed by an off time of 500 ms. The peak negative pressure was 13.5 ± 1.5 MPa measured in water with fiber optical hydrophone HFO090 (Onda Corporation, CA). The peak positive pressure was 65 ± 10 MPa. The *in situ* intraparenchymal temperature measurements with a 0.4-mm type T thermocouple showed a temperature rise between 3 and 3.5 °C when HIFU was applied. We sonicated 48 mice total in this study and did not observe any procedure-related mortality or serious adverse events. Likewise, we did not detect any noticeable change in daily ambulation or other maintenance activities such as grooming or climbing to obtain food and water.

### Histology

To assess neuroinflammatory responses of the brain to HIFU exposure, ultrasound pulses were applied through the scalp and skull at a position 1.5 mm lateral to the crossing of the midline between two eyes and the mid-way between eye and ear, approximately over the left primary motor cortex, as depicted in Fig. 1B. At 24 hours and 1 week after HIFU exposure or sham treatment, animals were anesthetized with sodium pentobarbital (100 mg/kg, i.p.) followed by transcardial perfusion of saline and then Formalin (10% phosphate buffer, Fisher Scientific). Mice then were decapitated, and the brain was removed and immersed in Formalin overnight. 50 µm coronal brain sections were acquired with a vibrating microtome at anteroposterior (AP) levels between + 1.5 mm and −2.5 mm. Slices at + 1, 0, −1, −2 mm Bregma were labeled with glial fibrillary acidic protein (GFAP) to assess astrogliosis, and ionized calcium-binding adaptor molecule (Iba1) to examine microglial reactivity, and neuronal nuclei (NeuN) to stain for neurons. For GFAP staining, floating slices were incubated overnight with monoclonal anti-mouse GFAP antibody (1:500 dilution, Invitrogen, Catalog # A-21282). The slices were then incubated with Peroxidase-conjugated AffiniPure goat anti-rat IgG (H + L) antibody (1:100 dilution, Jackson ImmunoResearch Laboratories, Catalog # 112–035–143) and stained with a 3,3-diaminobenzidine horseradish peroxidase substrate kit (Vector Laboratories, Catalog # SK-4100), then mounted onto gelatin subbed slides and coverslipped. For Iba1 staining, a rabbit polyclonal Iba1 antibody (1:500, Wako, Catalog # 019-19741) was used as primary antibody, and Peroxidase-conjugated AffiniPure goat anti-rabbit IgG (1:500 dilution, Jackson ImmunoResearch Laboratories, Catalog # 111-035-144) as secondary antibody. For NeuN staining, a chicken polyclonal antibody (1:500, Millipore, Catalog ABN91) was used as the primary antibody, and Peroxidase conjugated AffiniPure goat anti-chicken IgY (1:500, Jackson ImmunoResearch, Catalog # 103-035-155) was the secondary.

Prussian blue staining was done on brain slices mounted and dried on gelatin subbed slides. Mounted slides were dipped in deionized water then into the prepared Iron Stain for 3 minutes. The iron stain constituted of an equal mixture of 2% hydrochloric acid (Iron stain kit, ab150674, Abcam, Cambridge, MA) and potassium ferrocyanide solution (Iron stain kit). Slides were rinsed once more in deionized water to wash off the excess iron stain following counter stain with Nuclear Fast Red Solution (Iron stain kit). Slides were dehydrated in increasing concentrations of ethanol and xylene. Paramount was used to coverslip and slides were left to dry for at least 24 hours.

10 X images were taken using Zeiss Axio bright field microscope (Germany). ImageJ software was used to measure cell density following region of interest selection (Fig. 2B).

### Image analysis

Quantitative image analysis was completed using ImageJ software (National Institutes of Health) (Fig. 2A) and MATLAB (MathWorks). Anatomical regions of interest (ROIs) were manually drawn in ImageJ to subdivide coronal slices into identifiable brain structures. Images were first preprocessed using background subtraction and thresholding functions in ImageJ. The watershed plugin was used to split overlapping cell areas and Analyze Particles was used to identify cells conforming to a specific size. Percent area was then measured for each individual ROI.

### Rotarod and open field test (OFT)

A single station rotarod treadmill (Med Associates Inc.) was used for the rotarod test. The treadmill was designed for mouse with a 3.2 cm diameter shaft, 5.7 cm lane width, 16.5 cm fall height, and 24.8 cm diameter lane divider. All experimental mice received training for three days consisting of three 5 min trials with about 15 min intervals. The rotating speed of the rod was preset at 24 rpm for training. Following training, the mice were split into two groups and given either Sham or HIFU at random. Sham animals were subjected to the same operations (anesthesia, hair removal, HIFU apparatus in place etc.) as HIFU animals, but without ultrasound pulse application. Rotarod test, five 3 min trials with 3 min interval at 32 rpm, was performed at 2 hour, 24 hour, 1 week and 1 month after HIFU or sham treatment. Locomotor abilities of the mouse were quantitatively analyzed by comparing the latency that the mouse remained on the rod. Locomotor capabilities in sham animals were lower at 1 month as compared to acute timepoints, likely due to the pre-conditioning all groups received prior to HIFU application.

OFT was conducted 2 hour, 24 hour, 1 week and 1 month after either sham or HIFU exposure. Before the test, animals were brought to the testing room with dim ambient light for ~1 hour for adaptation. For the test, animals were placed in a square arena (40 cm × 40 cm × 30 cm; W X L X H) with a glass floor (Free Walk Box, CleverSys). Animals’ movements were recorded with a video camera from the bottom, and tracked and stored with FreeWalkScan software (CleverSys) for 20 mins. Post-hoc behavioral analysis, including distance travelled, freezing, and time spent in the center, was performed with customized Matlab code (Mathworks).

### Electrophysiology data acquisition

To characterize effect of HIFU on brain surface activities, we used µECoG arrays (Neuronexus) with 16 small recording electrodes (surface area ~0.03 mm^2^) arranged in a 4 × 4 grid, allowing us to sample small populations of neurons from the superficial layers of cortex at high resolution. Epidural neural signals were acquired with a NeuraLynx Digital Data Acquisition System, including a DL 4SX-M 32ch Base, a headstage pre-amplifier HS-18-CNR-MDR50, a HS-18-N2T-16 adaptor and Cheetah data acquisition software, at a sampling frequency of 16 kHz from freely moving animals in their home cages. High pass filtered (HPF; 900 to 5 kHz) and ECoG (0.1 and 300 Hz) signals were saved with Cheetah software (NeuraLynx). The animals’ behavioral state was simultaneously recorded and classified into Move or Resting status with HomeCageScan software (CleverSys) during electrophysiological recordings.

Ipsilateral and contralateral ECoG data were collected from different cohorts of animals. Contralateral animals were allowed to recover from implantation surgery for a week, then four-week baseline data were collected (Fig. 1A, n = 10 in both HIFU and sham groups). In ipsilateral cohort, animals were subjected to HIFU/sham treatment immediately before ECoG array implantation surgery, therefore, no baseline data were collected (Fig. 1A in purple, n = 4 in both HIFU and sham groups).

### Electrophysiology data processing

ECoG and HPF signals and home-cage movement status data were imported into Matlab (Mathworks) for manual removal of ECoG segments with myoelectric and/or movement artifacts (Fig. 1C). ECoG signals were down-sampled to 2 kHz, and then scored for the presence of artifacts based on the HPF signal and the animal’s movement state. Only ECoG snippets with ≥ 4 s artifact-free data, and recording sessions with ≥ 10 clean snippets, were used for quantitative analysis. We observed a close correlation between animal’s movement status and presence of artifacts (Fig. 1C), and the data used for analysis was primarily from the quiescent state. Power spectral density (PSD) for each recording session was computed with a multitaper Fast Fourier Transform (FFT) (NW = 3, k = 5) estimate using Matlab functions from the Chronux Toolkit^79^. Absolute and relative power were obtained for the δ (1–4 Hz), ɵ (4–8 Hz), α (8–13 Hz), β (13–30 Hz) and γ (30–100 Hz) frequency bands. Relative power was calculated by dividing power at certain frequency by the total power between 1 and 100 Hz. To exclude the possible effect of 60 Hz noise on the power estimation, signals between 56 and 64 Hz were removed. Coherence between all pairwise combinations of electrodes was also computed with Matlab functions from the Chronux Toolkit^79^. Sample entropy was calculated with the *SampEn* function in Matlab (Mathworks). To control for the state of the animal during the recordings, we compared the ECoG outcome measurements and the length of quiescence for every animal. We found no correlation between the length of quiescent recordings and the ECoG parameters. (r ≤ 0.23; n = 641 recording sessions, Pearson’s correlation).

### Statistics

Nonparametric Wilcoxon rank sum test followed by Benjamin and Hochberg false discovery rate correction was performed to statistically compare HIFU and sham groups in the density of glial reactions in each brain region (Fig. 2). Kolmogorov-Smirnov test was used to compare the probability distribution in the density of glial reactions across brain regions. When comparing differences between HIFU and sham groups with longitudinal measurements, we used two-way mixed-effect model with Sidak’s multiple comparison correction (GraphPad Prism) (Figs 3, 5, 7 and 8). One-way Friedman and Tukey’s tests were used to compare changes from baseline (Figs 4, 7 and S1). Wilcoxon rank sum test was used to compare HIFU and sham groups in other conditions. We used more conservative nonparametric test in this study because of the relatively small sample size, which makes normality test less reliable. All data are expressed as mean ± SE.

### Disclaimer

The mention of commercial products, their sources, or their use in connection with material reported herein is not to be construed as either an actual or implied endorsement of such products by the Department of Health and Human Services.

## Supplementary information

Supplemental Figures

## References

[1] Yoo SS (2011). Focused ultrasound modulates region-specific brain activity. Neuroimage.

[2] Verhagen, L. *et al*. Offline impact of transcranial focused ultrasound on cortical activation in primates. *Elife* **8**, 10.7554/eLife.40541 (2019). 10.7554/eLife.40541 PMC6372282 30747105

[3] Dallapiazza RF (2018). Noninvasive neuromodulation and thalamic mapping with low-intensity focused ultrasound. J Neurosurg.

[4] Lee H (2008). Focal lesions in acute mild traumatic brain injury and neurocognitive outcome: CT versus 3T MRI. J Neurotrauma.

[5] Grossman EJ, Inglese M, Bammer R (2010). Mild traumatic brain injury: is diffusion imaging ready for primetime in forensic medicine?. Top Magn Reson Imaging.

[6] Davis GA, Iverson GL, Guskiewicz KM, Ptito A, Johnston KM (2009). Contributions of neuroimaging, balance testing, electrophysiology and blood markers to the assessment of sport-related concussion. Br J Sports Med.

[7] Rezayat E, Toostani IG (2016). A Review on Brain Stimulation Using Low Intensity Focused. Ultrasound. Basic and clinical neuroscience.

[8] Fisher JA (2016). Real-Time Detection and Monitoring of Acute Brain Injury Utilizing Evoked Electroencephalographic Potentials. IEEE Trans Neural Syst Rehabil Eng.

[9] Kim H (2015). Suppression of EEG visual-evoked potentials in rats through neuromodulatory focused ultrasound. Neuroreport.

[10] Huang S (2018). Epidermal Electrode Technology for Detecting Ultrasonic Perturbation of Sensory Brain Activity. IEEE Trans Biomed Eng.

[11] Boutet A (2018). Focused ultrasound thalamotomy location determines clinical benefits in patients with essential tremor. Brain.

[12] McCabe JT (2014). Application of high-intensity focused ultrasound to the study of mild traumatic brain injury. Ultrasound in medicine & biology.

[13] Maxwell AD (2009). Noninvasive thrombolysis using pulsed ultrasound cavitation therapy - histotripsy. Ultrasound Med Biol.

[14] Pajek D, Burgess A, Huang Y, Hynynen K (2014). High-intensity focused ultrasound sonothrombolysis: the use of perfluorocarbon droplets to achieve clot lysis at reduced acoustic power. Ultrasound Med Biol.

[15] Ahadi G (2013). Transcranial sonothrombolysis using high-intensity focused ultrasound: impact of increasing output power on clot fragmentation. J Ther Ultrasound.

[16] Yang W, Zhou Y (2017). Effect of pulse repetition frequency of high-intensity focused ultrasound on *in vitro* thrombolysis. Ultrason Sonochem.

[17] Wright C, Hynynen K, Goertz D (2012). *In vitro* and *in vivo* high-intensity focused ultrasound thrombolysis. Invest Radiol.

[18] Suo D, Guo S, Lin W, Jiang X, Jing Y (2015). Thrombolysis using multi-frequency high intensity focused ultrasound at MHz range: an *in vitro* study. Phys Med Biol.

[19] Holscher T (2013). Effects of varying duty cycle and pulse width on high-intensity focused ultrasound (HIFU)-induced transcranial thrombolysis. J Ther Ultrasound.

[20] Goldstein LE (2012). Chronic traumatic encephalopathy in blast-exposed military veterans and a blast neurotrauma mouse model. Sci Transl Med.

[21] Gale SD, Johnson SC, Bigler ED, Blatter DD (1995). Nonspecific white matter degeneration following traumatic brain injury. J Int Neuropsychol Soc.

[22] Gale SD, Prigatano GP (2010). Deep white matter volume loss and social reintegration after traumatic brain injury in children. J Head Trauma Rehabil.

[23] Baumann CR (2009). Loss of hypocretin (orexin) neurons with traumatic brain injury. Ann Neurol.

[24] Levin HS (1990). Corpus callosal atrophy following closed head injury: detection with magnetic resonance imaging. J Neurosurg.

[25] Malkesman O, Tucker LB, Ozl J, McCabe JT (2013). Traumatic brain injury - modeling neuropsychiatric symptoms in rodents. Front Neurol.

[26] Mott TF, McConnon ML, Rieger BP (2012). Subacute to chronic mild traumatic brain injury. Am Fam Physician.

[27] Hamm RJ, Pike BR, O’Dell DM, Lyeth BG, Jenkins LW (1994). The rotarod test: an evaluation of its effectiveness in assessing motor deficits following traumatic brain injury. J Neurotrauma.

[28] Awwad HO (2016). Detecting Behavioral Deficits Post Traumatic Brain Injury in Rats. Methods Mol Biol.

[29] Meabon JS (2016). Repetitive blast exposure in mice and combat veterans causes persistent cerebellar dysfunction. Sci Transl Med.

[30] Koob AO, Colby JM, Borgens RB (2008). Behavioral recovery from traumatic brain injury after membrane reconstruction using polyethylene glycol. J Biol Eng.

[31] Buzsaki G, Schomburg EW (2015). What does gamma coherence tell us about inter-regional neural communication?. Nature neuroscience.

[32] Panagiotou M, Vyazovskiy VV, Meijer JH, Deboer T (2017). Differences in electroencephalographic non-rapid-eye movement sleep slow-wave characteristics between young and old mice. Sci Rep.

[33] Choi JJ, Pernot M, Small SA, Konofagou EE (2007). Noninvasive, transcranial and localized opening of the blood-brain barrier using focused ultrasound in mice. Ultrasound in medicine & biology.

[34] Nightingale KR (2015). Conditionally Increased Acoustic Pressures in Nonfetal Diagnostic Ultrasound Examinations Without Contrast Agents: A Preliminary Assessment. J Ultrasound Med.

[35] Wahab RA (2012). Mechanical bioeffects of pulsed high intensity focused ultrasound on a simple neural model. Med Phys.

[36] Goss SA, Frizzell LA, Dunn F (1979). Ultrasonic absorption and attenuation in mammalian tissues. Ultrasound in medicine & biology.

[37] Acosta C, Anderson HD, Anderson CM (2017). Astrocyte dysfunction in Alzheimer disease. J Neurosci Res.

[38] Donat CK, Scott G, Gentleman SM, Sastre M (2017). Microglial Activation in Traumatic Brain Injury. Front Aging Neurosci.

[39] Hernandez-Ontiveros DG (2013). Microglia activation as a biomarker for traumatic brain injury. Front Neurol.

[40] Pekny M, Wilhelmsson U, Tatlisumak T, Pekna M (2019). Astrocyte activation and reactive gliosis-A new target in stroke?. Neuroscience letters.

[41] Mahan Margaret Y., Thorpe Maxwell, Ahmadi Aliya, Abdallah Tessneem, Casey Hannah, Sturtevant Dylan, Judge-Yoakam Sénait, Hoover Caleb, Rafter Daniel, Miner James, Richardson Chad, Samadani Uzma (2019). Glial Fibrillary Acidic Protein (GFAP) Outperforms S100 Calcium-Binding Protein B (S100B) and Ubiquitin C-Terminal Hydrolase L1 (UCH-L1) as Predictor for Positive Computed Tomography of the Head in Trauma Subjects. World Neurosurgery.

[42] Tumer N (2013). Overpressure blast-wave induced brain injury elevates oxidative stress in the hypothalamus and catecholamine biosynthesis in the rat adrenal medulla. Neuroscience letters.

[43] Shively SB (2016). Characterisation of interface astroglial scarring in the human brain after blast exposure: a post-mortem case series. The Lancet. Neurology.

[44] Rubovitch V (2011). A mouse model of blast-induced mild traumatic brain injury. Exp Neurol.

[45] Sato T, Shapiro MG, Tsao DY (2018). Ultrasonic Neuromodulation Causes Widespread Cortical Activation via an Indirect Auditory Mechanism. Neuron.

[46] Guo H (2018). Ultrasound Produces Extensive Brain Activation via a Cochlear Pathway. Neuron.

[47] Arciniegas DB (2011). Clinical electrophysiologic assessments and mild traumatic brain injury: state-of-the-science and implications for clinical practice. Int J Psychophysiol.

[48] Gaetz M, Bernstein DM (2001). The current status of electrophysiologic procedures for the assessment of mild traumatic brain injury. J Head Trauma Rehabil.

[49] Gosselin N (2012). Evaluating the cognitive consequences of mild traumatic brain injury and concussion by using electrophysiology. Neurosurg Focus.

[50] Haneef Z, Levin HS, Frost JD, Mizrahi EM (2013). Electroencephalography and quantitative electroencephalography in mild traumatic brain injury. J Neurotrauma.

[51] Nuwer MR, Hovda DA, Schrader LM, Vespa PM (2005). Routine and quantitative EEG in mild traumatic brain injury. Clin Neurophysiol.

[52] Rapp PE (2015). Traumatic brain injury detection using electrophysiological methods. Front Hum Neurosci.

[53] Modarres, M., Kuzma, N. N., Kretzmer, T., Pack, A. I. & Lim, M. M. EEG slow waves in traumatic brain injury: Convergent findings in mouse and man. *Neurobiology of sleep and circadian rhythms* **1** (2016). PMC5175467 28018987

[54] Munia TTK, Haider A, Schneider C, Romanick M, Fazel-Rezai R (2017). A Novel EEG Based Spectral Analysis of Persistent Brain Function Alteration in Athletes with Concussion History. Sci Rep.

[55] Franke LM, Walker WC, Hoke KW, Wares JR (2016). Distinction in EEG slow oscillations between chronic mild traumatic brain injury and PTSD. Int J Psychophysiol.

[56] Thatcher RW, Biver C, McAlaster R, Camacho M, Salazar A (1998). Biophysical linkage between MRI and EEG amplitude in closed head injury. Neuroimage.

[57] Tebano MT (1988). EEG spectral analysis after minor head injury in man. Electroencephalography and clinical neurophysiology.

[58] Korn A, Golan H, Melamed I, Pascual-Marqui R, Friedman A (2005). Focal cortical dysfunction and blood-brain barrier disruption in patients with Postconcussion syndrome. Journal of clinical neurophysiology: official publication of the American Electroencephalographic Society.

[59] Lim MM (2013). Dietary therapy mitigates persistent wake deficits caused by mild traumatic brain injury. Sci Transl Med.

[60] Vyazovskiy VV (2011). Local sleep in awake rats. Nature.

[61] Hung CS (2013). Local experience-dependent changes in the wake EEG after prolonged wakefulness. Sleep.

[62] Imbach LL (2015). Increased sleep need and daytime sleepiness 6 months after traumatic brain injury: a prospective controlled clinical trial. Brain: a journal of neurology.

[63] Pesaran B, Pezaris JS, Sahani M, Mitra PP, Andersen RA (2002). Temporal structure in neuronal activity during working memory in macaque parietal cortex. Nature neuroscience.

[64] Gregoriou GG, Gotts SJ, Zhou H, Desimone R (2009). High-frequency, long-range coupling between prefrontal and visual cortex during attention. Science.

[65] Rouhinen S, Panula J, Palva JM, Palva S (2013). Load dependence of beta and gamma oscillations predicts individual capacity of visual attention. The Journal of neuroscience: the official journal of the Society for Neuroscience.

[66] Welle CG, Contreras D (2016). Sensory-driven and spontaneous gamma oscillations engage distinct cortical circuitry. J Neurophysiol.

[67] Welle CG, Contreras D (2017). New Light on Gamma Oscillations. Neuron.

[68] Dunkley BT (2015). Low-frequency connectivity is associated with mild traumatic brain injury. Neuroimage Clin.

[69] Kumar S, Rao SL, Chandramouli BA, Pillai SV (2009). Reduction of functional brain connectivity in mild traumatic brain injury during working memory. J Neurotrauma.

[70] Rigon A, Duff MC, McAuley E, Kramer AF, Voss MW (2016). Is Traumatic Brain Injury Associated with Reduced Inter-Hemispheric Functional Connectivity? A Study of Large-Scale Resting State Networks following Traumatic Brain Injury. J Neurotrauma.

[71] Liang Z (2015). EEG entropy measures in anesthesia. Frontiers in computational neuroscience.

[72] Tong S, Bezerianos A, Malhotra A, Zhu Y, Thakor N (2003). Parameterized entropy analysis of EEG following hypoxic-ischemic brain injury. Physics Letters A.

[73] Gosseries O (2011). Automated EEG entropy measurements in coma, vegetative state/unresponsive wakefulness syndrome and minimally conscious state. Funct Neurol.

[74] DePalma, R. G. In *Brain Neurotrauma: Molecular*, *Neuropsychological*, *and Rehabilitation Aspects Frontiers in* Neuroengineering (ed Kobeissy, F. H.) (2015). 26269865

[75] Mac Donald CL (2011). Detection of blast-related traumatic brain injury in U.S. military personnel. N Engl J Med.

[76] Einarsen, C. *et al*. Patients with Mild Traumatic Brain Injury Recruited from both Hospital and Primary Care Settings: a Controlled Longitudinal MRI Study. *J Neurotrauma*, 10.1089/neu.2018.6360 (2019). 10.1089/neu.2018.6360 PMC6818486 31280698

[77] Polikov VS, Tresco PA, Reichert WM (2005). Response of brain tissue to chronically implanted neural electrodes. J Neurosci Methods.

[78] Jorfi M, Skousen JL, Weder C, Capadona JR (2015). Progress towards biocompatible intracortical microelectrodes for neural interfacing applications. J Neural Eng.

[79] Bokil H, Andrews P, Kulkarni JE, Mehta S, Mitra PP (2010). Chronux: a platform for analyzing neural signals. J Neurosci Methods.

