# Supplementary material for: Longitudinal Functional Assessment of Brain Injury Induced by High-Intensity Ultrasound Pulse Sequences
Source: Sci Rep. 2019 Oct 29;9:15518. doi: 10.1038/s41598-019-51876-5 (PMC6820547; doi:10.1038/s41598-019-51876-5)
Supplement: Supplementary file 1 — Supplemental Figures [file 41598_2019_51876_MOESM1_ESM.pdf]

Supplemental Figures

Longitudinal Functional Assessment of Brain Injury Induced by High-Intensity Ultrasound Pulse Sequences

Authors: Meijun Ye[1*], Krystyna Solarana[1], Harmain Rafi[1], Shyama Patel[1,2], Marjan Nabili[3,4], Yunbo Liu[3], Stanley Huang[1], Jonathan A.N. Fisher[1,5], Victor Krauthamer[1], Matthew Myers[3], and Cristin Welle[1,6*]

[1] Division of Biomedical Physics, Office of Science and Engineering Laboratories, Center for Devices and Radiological Health, Food and Drug Administration, Silver Spring, MD

[2] Division of Neurological and Physical Medicine Devices, Office of Device Evaluation, Center for Devices and Radiological Health, Food and Drug Administration, Silver Spring, MD

[3] Division of Applied Mechanics, Office of Science and Engineering Laboratories, Center for Devices and Radiological Health, Food and Drug Administration, Silver Spring, MD

[4] Division of Radiological Health, Office of In Vitro Diagnostics and Radiological Health, Center for Devices and Radiological Health, Food and Drug Administration, Silver Spring, MD

[5] Department of Physiology, New York Medical College, Valhalla, NY

[6] Departments of Neurosurgery and Physiology & Biophysics, University of Colorado Anschutz Medical Campus, Aurora, CO

[*] Correspondence: Cristin.welle@cuanschutz.edu and Meijun.ye@fda.hhs.gov

Supplemental Figures

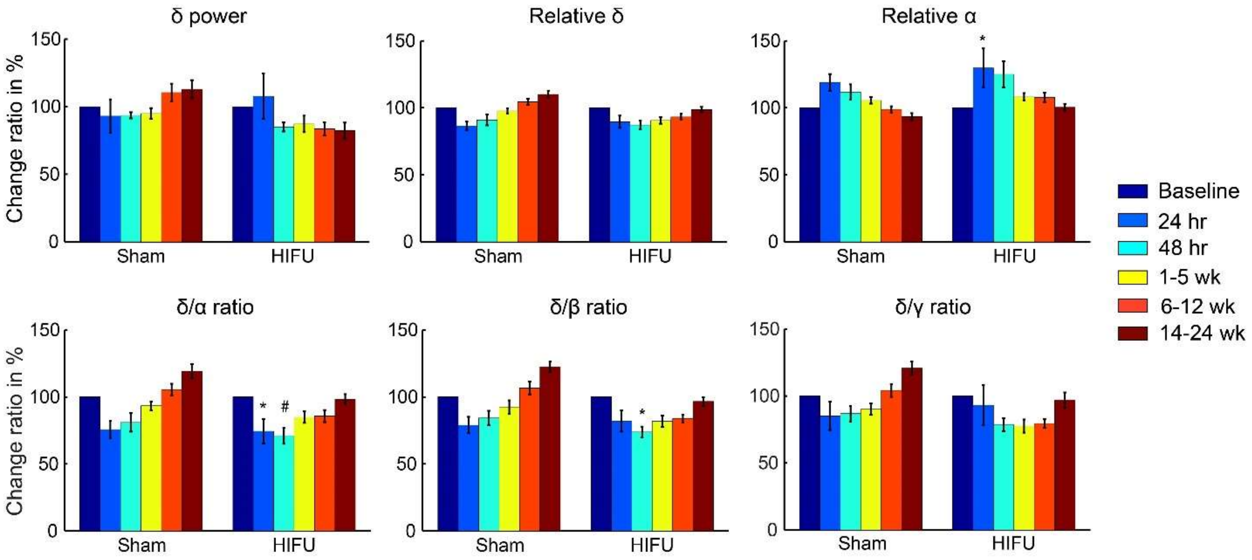

Supplemental Figure 1. Longitudinal power change from the baseline on the contralateral side to HIFU. Though 10 animals were recorded in each group at each time point, only 6 animals in HIFU group and 3 animals in sham group were included in Friedman and post-hoc Tukey tests due to insufficient clean ECoG traces in some recordings at either 24 or 48 hours post-treatment (see Methods for criteria). Only ECoG parameters with $p<0.1$ in Friedman test are shown in the figure. (Data expressed as mean ± se, # $p<0.1$, * $p < 0.05$, Tukey test)

Supplemental Figures

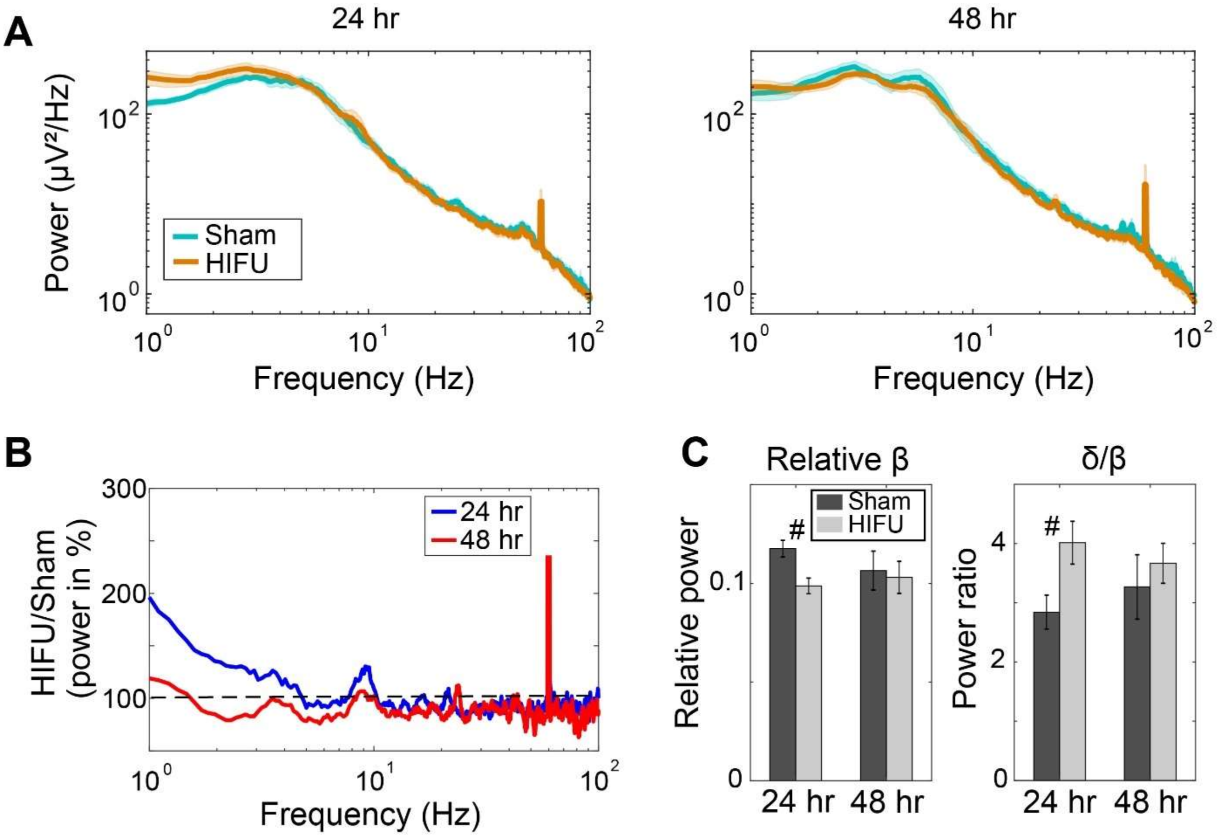

Supplemental Figure 2. Acutely altered PSD after HIFU on the contralateral hemisphere compared to sham. A. PSD at 24 and 48 hours post sham treatment or HIFU. **B.** Difference in the PSD between sham and injured groups at 24 and 48 hours post treatment. **C.** Injured mice had significantly lower relative β and higher δ/β ratio at 24 hours post HIFU compared to sham animals. (In sham group, n=7 at 24 hours post-treatment, and n=4 at 48 hours post-treatment. In injured group, n=8 at both 24 and 48 hours post-treatment. # $p < 0.1$, Two-way mixed-effects model followed by Sidak's multiple comparison test [GraphPad Prism])

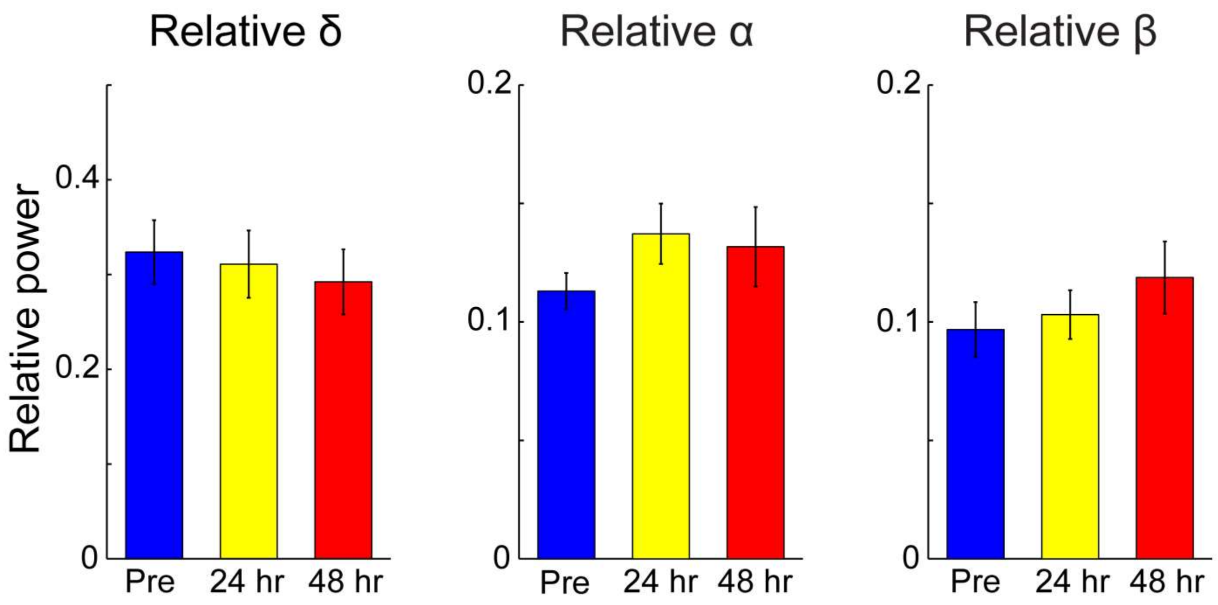

Supplemental Figure 3. Acute PSD changes after 30 mins isoflurane exposure. (n=4)

Supplemental Figures

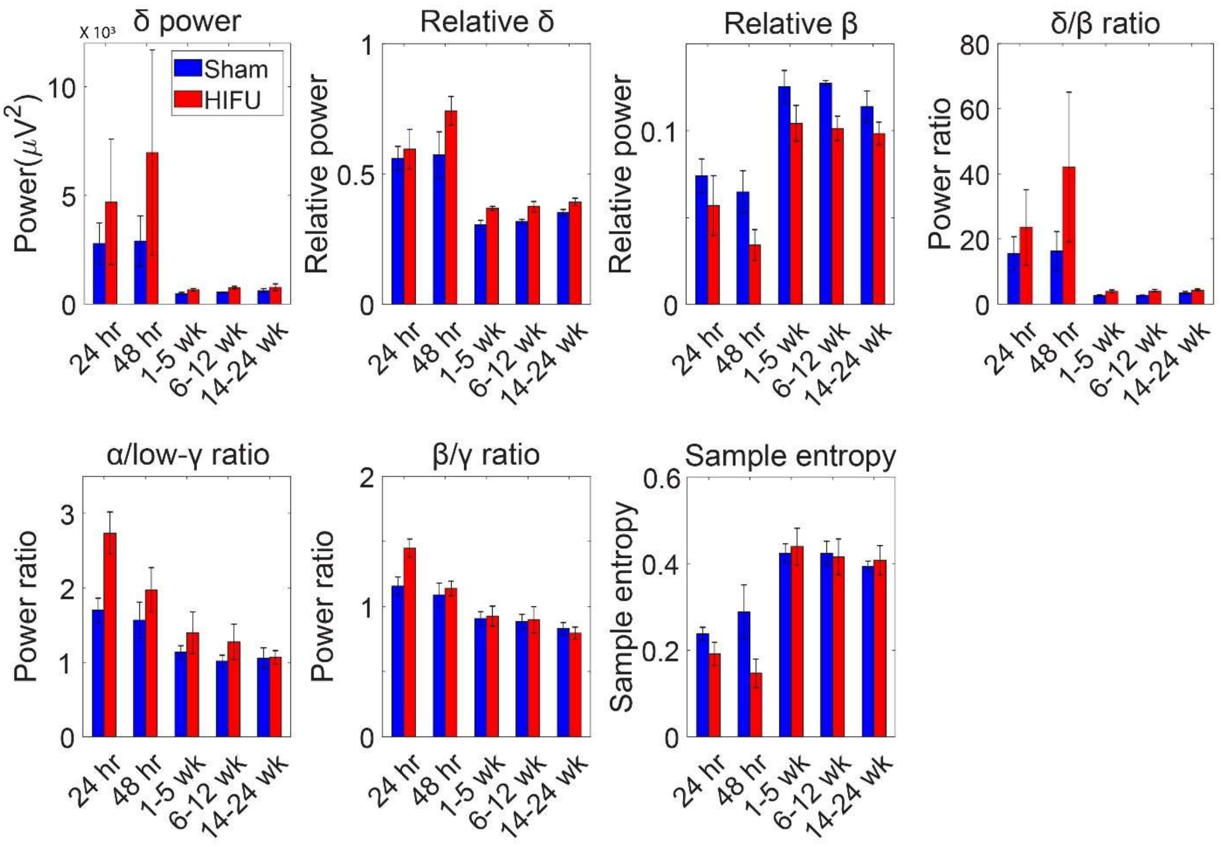

Supplemental Figure 4. Longitudinal ECoG signal change on the ipsilateral hemisphere to HIFU.

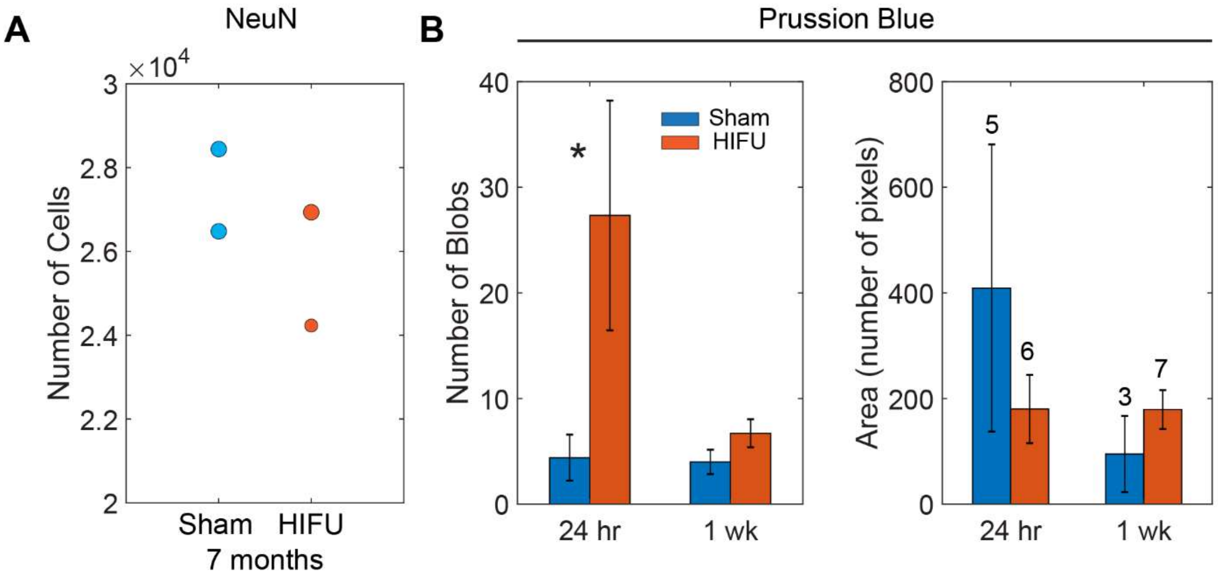

Supplemental Figure 5. Histological evaluation of tissue reactions after HIFU exposure. A. NeuN staining 7 months after treatments. Four animals subjected to rotarod test were kept alive for 7 months after treatment, then NeuN staining was performed. Density of NeuN staining in HIFU exposed animals was lightly lower than sham animals at Bregma 0. However, the difference is not remarkable enough to conclude neuronal cell death at chronic time point. **B.** Prussian Blue Staining at 24 hours and 1 week after treatment. The number of iron spots was significantly higher in HIFU exposed animals than sham animals at 24 hours post treatment. However, the differences were not significant at 1 week group, neither the area of Prussian Blue. (Wilcoxon rank sum test, * $p < 0.05$)
